# Unveiling the common mechanisms and therapeutic targets of medicinal herbs in acute pancreatitis: a network pharmacology and experimental validation approach

**DOI:** 10.1186/s40643-025-00925-1

**Published:** 2025-07-30

**Authors:** Yuxin Shi, Yan Jia, Ya Liu, Hanyue Wang, Honghui Liu, Yilin Huang, Peiyan Chen, Jie Peng

**Affiliations:** 1 Department of Gastroenterology, Xiangya Hospital, Central South University, Changsha, 410008 Hunan China; 2 National Clinical Research Center for Geriatric Disorders, Xiangya Hospital, Central South University, Changsha, 410008 Hunan China

**Keywords:** Acute pancreatitis, Medicinal herbs, Network pharmacology, Linarin, PI3K/AKT signaling

## Abstract

**Background:**

Acute pancreatitis (AP) is an acute abdominalgia with complicated pathogenesis and high mortality, which is lacking in specific means for clinical diagnosis and treatment. Currently, numerous traditional Chinese medicines have demonstrated remarkable efficacy in AP. Given their multi-target and multi-compound actions, we hypothesize that an underlying common mechanism may contribute to their therapeutic effects. This study aimed to identify key therapeutic targets and potential strategies for AP by investigating the shared pharmacological effects of medicinal plants through network pharmacology analysis and experimental validation.

**Methods:**

We systematically searched the literature for medicinal herbs that have been reported in AP treatment. Next, we utilized the TCMSP database to identify active compounds that were present in at least two medicinal herbs. Key active compounds and targets were determined through Cytoscape analysis and a PPI network, followed by KEGG pathway enrichment analysis. Combined the core targets identified by Cytoscape and the targets enriched in the PI3K/AKT signaling pathway, molecular docking was performed to assess the binding affinity between the intersecting targets and active compounds. Finally, high-affinity compounds were screened, and linarin’s optimal binding profile led to its selection for further in vivo and in vitro experimental validation.

**Results:**

A total of 37 medicinal herbs were retrieved from the literature search. We identified 62 common compounds and 968 targets from medicinal herbs, further taking intersection to 319 targets for anti-AP. Based on this, “compound-target” and “target” networks were constructed, and the top 12 key active compounds and 11 targets were selected. KEGG analysis indicated that the PI3K/AKT pathway might be closely related to pancreatic protection. Molecular docking results showed that linarin exhibited good binding affinity with all core intersecting targets, particularly with AKT1. Subsequently, both in vivo and in vitro experiments demonstrated that linarin could alleviate AP-induced pancreatic damage and systemic inflammation. To further validate the mechanistic involvement of PI3K/AKT signaling pathway, we employed the PI3K/AKT activator 740 Y-P, which was found to effectively reverse linarin-mediated downregulation of PI3K/AKT activation, thereby confirming the crucial role of this pathway in linarin’s protective effects.

**Conclusion:**

Exploring therapeutic strategies based on common mechanisms and targets may be an effective approach. This study revealed that linarin and AKT1 were potential therapeutic compounds and targets for AP in the preclinical stage, which could provide theoretical support and new insights for the drug discovery of AP.

**Graphical Abstract:**

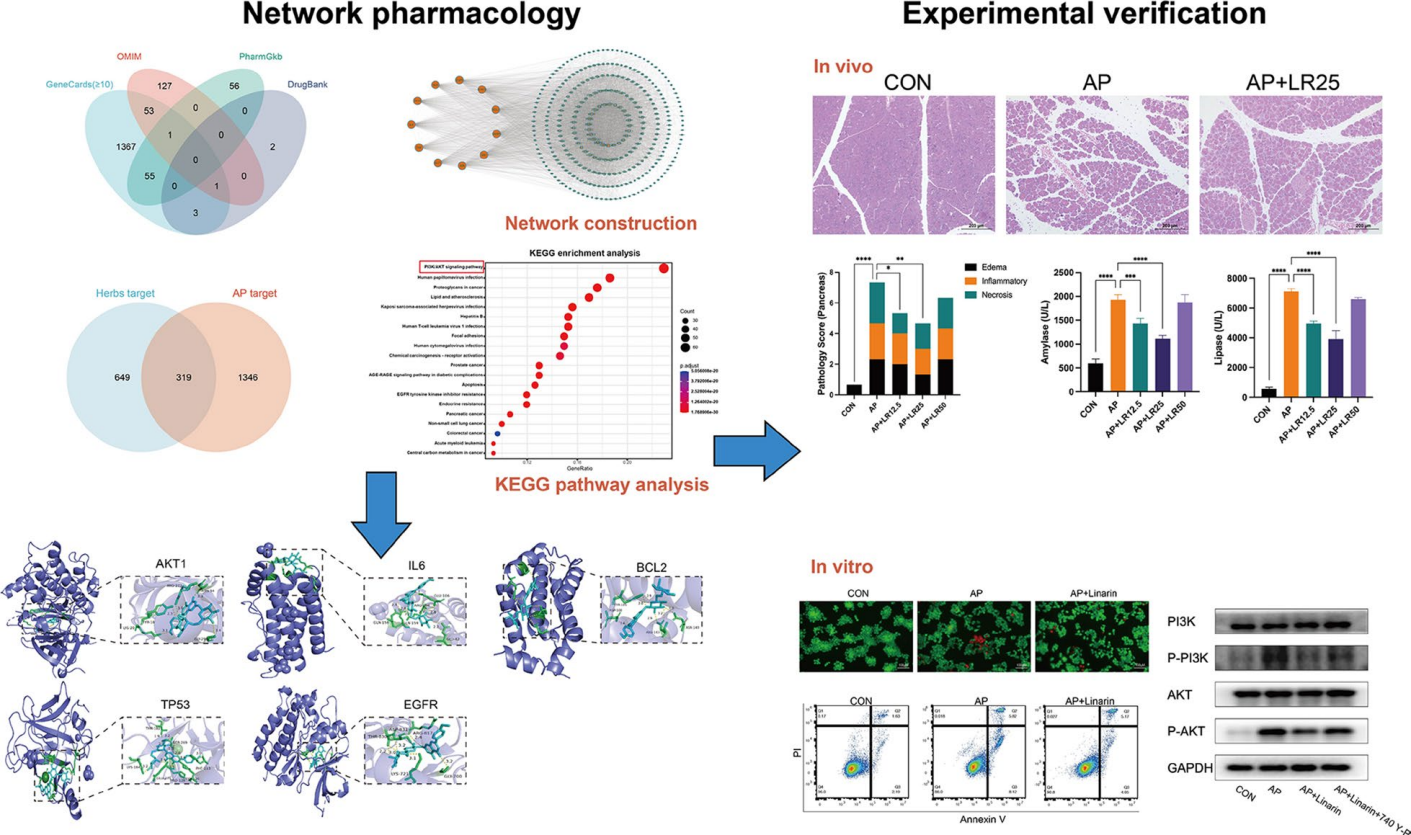

**Supplementary Information:**

The online version contains supplementary material available at 10.1186/s40643-025-00925-1.

## Introduction

Acute pancreatitis (AP) is one of the most common acute abdominal conditions, characterized by its elusive pathogenesis and highly variable clinical presentations (Gardner 2021). The incidence of AP varies globally, with a general rate of (23–49)/100,000 (Petrov and Yadav 2019). Despite recent advancements in diagnostic and therapeutic approaches, approximately 20% of patients still progress to severe acute pancreatitis (SAP), marked by persistent organ failure and pancreatic necrosis, with a mortality rate exceeding 30% (Banks et al. 2013). Currently, no specific therapeutic agents are available for AP, underscoring the urgent need for novel treatment options.

Traditional Chinese medicine (TCM), an integral part of Chinese medicine, has demonstrated promising clinical effects in AP treatment (Xiang et al. 2017). For thousands of years, TCM has retained its fundamental holistic approach, exerting therapeutic effects through multi-target, multi-compound actions. Numerous experimental studies on AP suggest that herbal medicines can exert consistent efficacy to reduce amylase and lipase levels, mitigate pancreatic histopathological damage, and alleviate inflammatory cell infiltration, which may be attributed to their targeting of common pathological mechanisms (Li et al. 2017). It has been reported that rhubarb ameliorates AP by inhibiting MAPK pathway activation and inflammatory mediator production (Feng et al. 2012). Similarly, ginkgo biloba extract has been found to alleviate AP by downregulating the TLR4/p38MAPK/JNK inflammatory signaling cascade (Mostafa et al. 2024). Similarly, Radix Salviae Miltiorrhizae and Curcuma longa have been demonstrated to offer a protective effect on AP by downregulating NF-κB (Yu et al. 2011; Zhang et al. 2010). Given the multi-compound and multi-target properties of TCM, it is hypothesized that shared pathological mechanisms may be closely associated with common active compounds and targets. Thus, a comprehensive analysis of shared active compounds and targets in TCM treatments for AP has the potential to provide a rational and effective strategy for AP treatment.

In recent years, the emergence of network pharmacology has provided new perspectives for constructing compound-target and target-disease networks, aligning well with the characteristics of TCM (van Hasselt and Iyengar 2019; Zhao and Iyengar 2012). This approach has already been successfully applied to various diseases, including AP, offering valuable tools for exploring novel therapeutic strategies and identifying specific therapeutic targets of TCM (Li et al. 2023b; Xu et al. 2024a; Zheng et al. 2024).

This study aimed to integrate relevant literature on medicinal herbs for AP treatment, utilizing network pharmacology and molecular docking strategies to investigate shared active compounds and core targets of these herbs against AP. Additionally, in vivo and in vitro experiments were conducted to validate the therapeutic potential and underlying mechanisms of the corresponding active compounds in AP.

## Materials and methods

### Collection of AP-effective medicinal herbs

The medicinal herbs reported in AP treatment were searched from PubMed, Embase, and Scopus databases. The literature including reviews and original articles was summarized, and then experienced manual filtering to exclude irrelevant literature and herbal formulas consisting of more than one type of medicinal herbs.

### Screening of active compounds and related targets based on network pharmacology

TCMSP database (https://www.tcmsp-e.com/#/database/) was utilized to screen active compounds of the medicinal herbs. The screening criteria were set as follows: drug-likeness (DL) ≥ 0.18 and oral bioavailability (OB) ≥ 30% (Li et al. 2025a; Sang et al. 2025), and the subsequent analysis comprehensively yielded the common compounds, which were then imported to the Swisstargetprediction database (http://swisstargetprediction.ch/) to find potential targets. For visualization, the common compounds and their targets were entered into Cytoscape 3.10.0 to construct the “compound-target” network diagram. Then, we selected “Network Analyzer” to implement the network topology analysis and screened the core active compounds through the “degree” value.

### Collection of the targets for AP

GeneCards database (https://www.genecards.org/), OMIM database (https://omim.org/), PharmGkb database (https://www.pharmgkb.org/), and DrugBank database (https://www.drugbank.ca/) were employed to acquire AP-related targets. Then, the intersection of herb targets and AP targets was obtained by Venny 2.1 (https://bioinfogp.cnb.csic.es/tools/venny/index.html).

### Network construction and core targets identification

The overlapping targets were imported to the STRING database (https://string-db.org/) to acquire the PPI network (confidence score > 0.4) and then visualized in Cytoscape 3.10.0 to obtain the “target” network. Then, CytoNCA plug-in was utilized to calculate the topological parameter, and core targets were identified under the “degree” value assessment.

### KEGG enrichment analysis and molecular docking

After carrying out the KEGG enrichment analysis through the Hiplot Pro platform (https://hiplot.com.cn/), the KEGG results and the topological analysis of the “compound-target” network as well as the “target” network were integrated to acquire core active compounds and targets to complement further molecular docking analysis. The structures of protein receptors and small molecule ligands were retrieved from the RCDB PDB database (https://www.rcsb.org/) and the PubChem database (https://pubchem.ncbi.nlm.nih.gov/), respectively. Chem3D 22.0.0 was utilized to minimize energy and transform the sdf file to mol2 file of small molecule ligands. Following the pretreatment of receptors and ligands through PyMOL and AutoDockTools (version 1.5.6), molecular docking was performed by AutoDock Vina, with visualization by PyMOL to assess the affinity of the interactions.

### Animals

6-week-old *C57BL/6J* mice were purchased from Hunan SJA Laboratory Animal Co., Ltd and raised in an SPF barrier environment with a standard diet and 12 h light/dark cycle. This experiment was approved by the Experimental Animal Ethics Committee, Xiangya Hospital, Central South University, China (approval number: 202503047). All the experimental protocols were in accordance with relevant guidelines.

### Experimental design

AP mouse model was induced by intraperitoneal injections of caerulein ten times, as previously reported (Jia et al. 2024). The mice were randomly divided into six groups: CON, AP, AP + 12.5 mg/kg linarin (AP + LR12.5), AP + 25 mg/kg linarin (AP + LR25), AP + 50 mg/kg linarin (AP + LR50) and 50 mg/kg linarin (LR50) groups. Linarin (HPLC ≥ 98%, B20860) was obtained from Shanghai Yuanye Bio-Technology Co., Ltd., dissolved in dimethyl sulfoxide (DMSO), and then suspended in 0.5% CMC-Na. The linarin groups were administrated with different concentrations of linarin for 7 days by gavage, while the other two groups received a corresponding 0.5% CMC-Na with DMSO. On day 7, the AP mouse model was constructed and the mice were euthanized to collect pancreatic tissues as well as serum samples.

### Hematoxylin and Eosin (HE) staining and immunohistochemistry staining

The pancreatic tissue was fixed in 4% paraformaldehyde, followed by embedding, sectioning, and deparaffinization. HE staining was then performed, and the sections were mounted for observation under a light microscope. The degree of pathological changes in the pancreas was evaluated based on previous studies (Feng et al. 2015). As for immunohistochemistry staining, antigen retrieval was performed using a boiling citrate solution after deparaffinization. Endogenous peroxidase was inactivated with 3% H₂O₂, followed by three washes with PBS and blocking with bovine serum albumin. Sections were incubated overnight with primary antibodies against CD68 and MPO, followed by incubation at room temperature with HRP-conjugated secondary antibodies. After DAB staining and hematoxylin counterstaining, slides were observed under a light microscope, and quantification was performed using ImageJ.

### Measurement of serological indicators

Serum samples were obtained by centrifuging at 12,000 rpm for 10 min, followed by measurement of serum amylase (Biosino, China) and lipase (Nanjing Jiancheng, China) levels according to the commercial kit instructions. To evaluate the toxicological effect of linarin on liver and kidney function in mice, we measured the levels of AST, ALT, BUN, and CREA in the 50 mg/kg Linarin group (LR50) and compared them with those in the CON group. To further assess the levels of inflammatory cytokines, IL-1β, IL-6, and TNF-α were measured by commercial kits (Jianglai Bio, China).

### TUNEL staining

Apoptotic cells in pancreatic tissue sections were detected using the TUNEL Apoptosis Detection Kit (Servicebio, China) following the manufacturer’s instructions. Briefly, pancreatic tissue sections of 4 μm thickness were deparaffinized, rehydrated, and treated with proteinase K at 37 °C for 20 min. After washing with PBS, sections were incubated with the reaction mixture in a humidified chamber at 37 °C for 1 h. After counterstaining with DAPI, the sections were visualized, and images were captured under a fluorescence microscope.

### Cell line and cell culture

AR42J cells were obtained from Zhong Qiao Xin Zhou Biotechnology Co.,Ltd (ZQ0145, China) and maintained in F12K medium supplemented with 20% fetal bovine serum (NEWZERUM, New Zealand) and 1% penicillin-streptomycin (Gibco, USA) at 37 °C in a humidified incubator with 5% CO₂. AR42J cells were treated with caerulein and linarin. The PI3K/AKT agonist 740 Y-P (20 µM, MedChemExpress, USA) was added to the cells to investigate the role of the PI3K/AKT pathway (Li et al. 2025b; Wang et al. 2024b). The cells were divided into: CON group, AP group, AP + Linarin group, and AP + Linarin + 740 Y-P group.

### Cell Counting Kit-8 (CCK-8) assay

AR42J cells were seeded in 96-well plates at an appropriate density and cultured for 24 h to allow adhesion. Subsequently, the cells were treated with linarin at varying concentrations (5, 10, 20, 40, 80, and 160 µM) for 24 h. Cell viability was assessed using the CCK-8 (Abbkine, China) following the manufacturer’s instructions. Briefly, 10 µL of CCK-8 reagent was added to each well, and the plates were incubated at 37 °C for 2 h. The absorbance was measured at 450 nm using a microplate reader (BioTek, USA). Linarin was dissolved in DMSO with ultrasonication to prepare a stock solution. The concentration and treatment duration of linarin were determined based on previous literature (Chen et al. 2017; Zhang et al. 2024).

### LDH release and Calcein Acetoxymethylester/Propidium Iodide (Calcein-AM/PI) staining assay

As previously described, an AP model was established by stimulating AR42J cells with 100 nM caerulein for 24 h (Song et al. 2022; Sun et al. 2022). AR42J cells were seeded in 96/12 well plates and randomly divided into the CON, AP, and linarin groups. Acinar cell death was assessed by measuring LDH release in the cell supernatant using the LDH Cytotoxicity Assay Kit (Beyotime, China) and by evaluating live/dead cells with the Calcein-AM/PI Double Staining Kit (Solarbio, China).

### Intracellular reactive oxygen species (ROS) measurement

The pretreated AR42J cells were incubated with 10 µM DCFH-DA (Abbkine, China) at 37 °C for 30 min. After incubation, the cells were washed 3 times with serum-free medium to remove excess dye. The fluorescence intensity was then observed under an inverted fluorescence microscope.

### Flow cytometric analysis of apoptosis and necrosis

Cell apoptosis and necrosis were evaluated using the Annexin V-FITC/PI Apoptosis Detection Kit (Meilunbio, China). Briefly, AR42J cells were seeded in 6-well plates and incubated with caerulein for 24 h in the presence or absence of linarin treatment. Then, cells were harvested, washed twice with pre-chilled PBS, and resuspended in 1×binding buffer. Subsequently, cells were stained with Annexin V-FITC (5 µL) and PI (10 µL) at room temperature in the dark for 15 min. Apoptotic and necrotic cell populations were analyzed using a Cytek NL3000 flow cytometer, and data were processed with FlowJo (version 10.8.1).

### Western blot

Total protein was extracted by adding RIPA lysis buffer with protease and phosphatase inhibitors. Protein concentration was determined using a BCA assay kit (Abbkine, China). Then, proteins were separated on a 10% SDS-PAGE gel and transferred to a PVDF membrane. The membrane was blocked with 3% BSA at room temperature for 1 h, followed by overnight incubation with primary antibodies: P-AKT, AKT, P-PI3K, and PI3K (diluted 1:1000). After incubation with corresponding secondary antibodies, bands were visualized using ECL detection reagents and quantified using ImageJ.

### Statistical analysis

Data were presented as mean ± standard deviation (SD) or median (interquartile range, IQR). Data normality was evaluated using the Shapiro-Wilk test. For comparisons between two groups, Student’s t-test was applied to normally distributed data, while the Mann-Whitney U-test was used for non-normal distributions. Multiple group comparisons were performed using either one-way analysis of variance (ANOVA, for parametric data) or the Kruskal-Wallis test (for non-parametric data). All statistical analyses and data visualization were performed using R (version 4.4.2) and GraphPad Prism (version 9.0). *P*-value < 0.05 was considered statistically significant.

## Result

### Acquisition of active compounds in medicinal herbs and target prediction

First, we retrieved a total of 37 medicinal herbs from PubMed, Embase, and Scopus databases, as listed in Table 1. Then, 518 compounds were screened through the TCMSP database, including 62 common compounds and 456 unique compounds (Table S1). Subsequently, we searched and eliminated duplication to obtain 968 targets of the 62 common compounds (Table S2).

**Table 1:** The information about the 37 medical herbs

Radix Salviae
Phyllanthi Fructus
Ginkgo Folium
Lithospermum Erythrorhizon
Gardeniae Fructus
Sennae Folium
Silybum Marianum
Herba Patriniae
Radix Rhei Et Rhizome
Curcumaelongae Rhizoma
Calendula Officinalis
Artemisia Annua L.
Sophorae Flavescentis Radix
Chuanxiong Rhizoma
licorice
Magnolia Officinalis Rehd Et Wils.
Piperis Fructus
Hedysarum Multijugum Maxim.
Scutellariae Radix
Chrysanthemi Indici Flos
Coptidis Rhizoma
Ginsen Radix Et Rhizoma Rubra
Epimrdii Herba
Radix Bupleuri
Herbahypericiperforati
Nardostachyos Radix Et Rhizoma
Gentianae Radix Et Rhozima
Lycii Fructus
Fructus Ligustri Lucidi
Centella Asiatica (L.) Urban[Hydro-Cotyle Asiatica L.]
Momordicae Fructus
Cichorii Radix
Croci Stigma
Kansui Radix
Spatholobus Suberectus Dunn
Evodiae Fructus
Hyperici Japonici Herber

### Targets of medicinal herbs in the treatment of AP

**Fig. 1:**
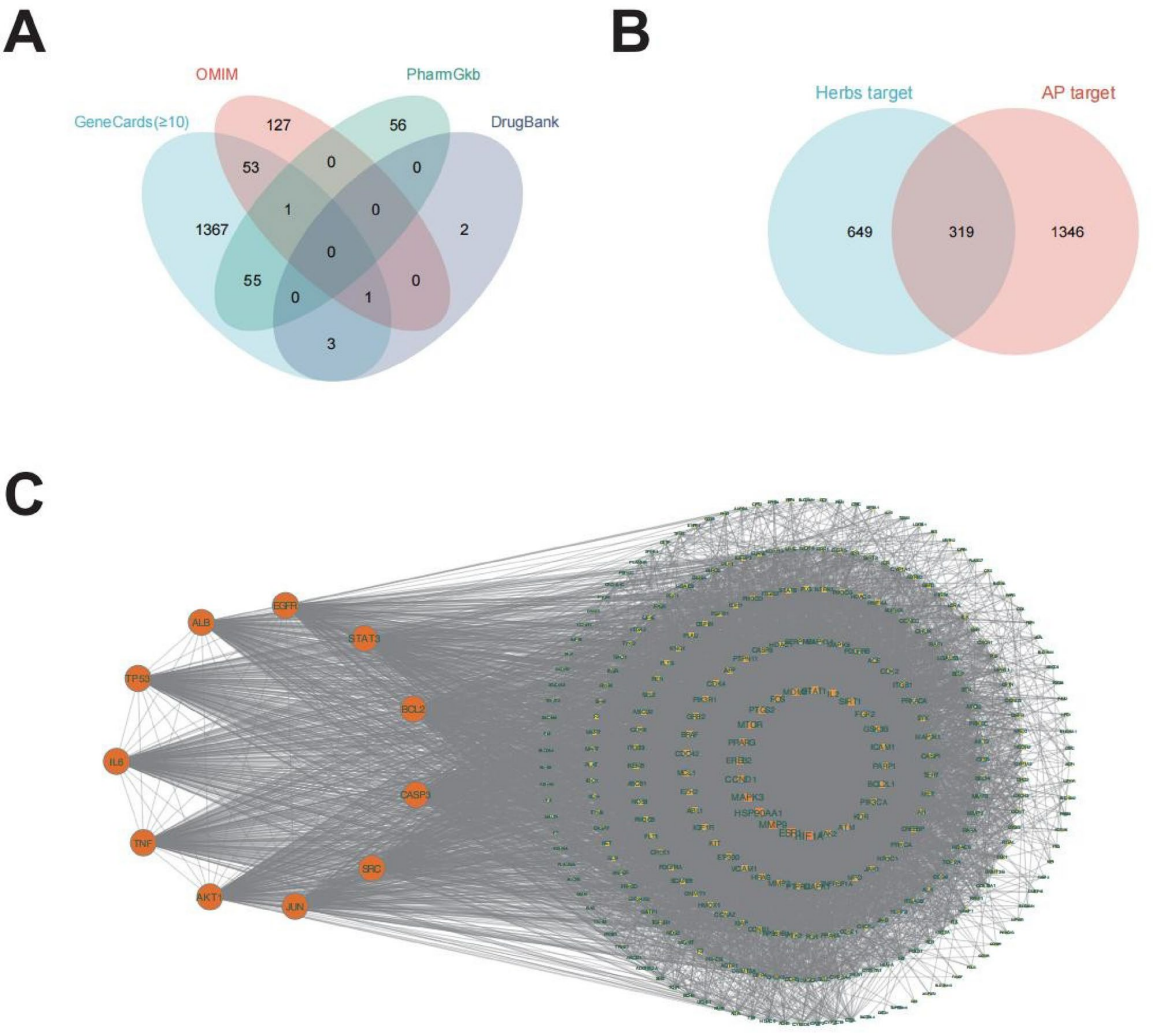
Targets of medicinal herbs in the treatment of AP. (**A**) Venn diagram of AP-related targets. (**B**) Venn diagram of the overlapping targets of medicinal herbs and AP. (**C**) Screening of core targets through the “target” network

We acquired a grand total of 1480 disease targets from the GeneCards database using a relevance score of ≥ 10. Out of these, 1665 targets were obtained, including 183 targets from the OMIM database, 112 targets from the PharmGkb database, and 6 targets from the DrugBank database (Table S3, Fig. 1A). To further clarify the specific mechanisms, we integrated the targets of medicinal herbs and AP. Finally, 319 common targets were obtained, as shown in Fig. 1B.

### Network construction and PPI analysis

We imported 62 common compounds and 968 targets into Cytoscape 3.10.0 to construct a compound-target network (Figure S1). The top 12 key active compounds are listed in Table 2, suggesting that these compounds might play an important role in AP treatment.

Next, a PPI network was constructed by importing the intersecting targets into the STRING database, which consisted of 318 nodes and 8495 edges. Using the CytoNCA plug-in, we identified the top 11 core targets (Fig. 1C), including AKT1 (degree = 217), TNF (degree = 205), IL6 (degree = 204), TP53 (degree = 202), ALB (degree = 194), EGFR (degree = 188), STAT3 (degree = 184), BCL2 (degree = 179), CASP3 (degree = 171), JUN (degree = 168), and SRC (degree = 168).

**Table 2:** The top 12 key active compounds

Compound name	PubChem CID	Degree
Mandenol	5,282,184	112
crocetin	5,281,232	111
Emodin-1-O-beta-D-glucopyranoside	5,319,333	110
licochalcone a	5,318,998	108
Vestitol	92,503	108
Linarin	5,317,025	107
24-methylidenelophenol	5,283,640	107
Obacunone	119,041	107
Ethyl oleate (NF)	5,363,269	106
(2R)-5,7-dihydroxy-2-(4-hydroxyphenyl)chroman-4-one	667,495	106
Linoleyl acetate	5,319,042	106
Sennoside E_qt	73,425,506	106

### KEGG enrichment and molecular docking analysis

**Fig. 2:**
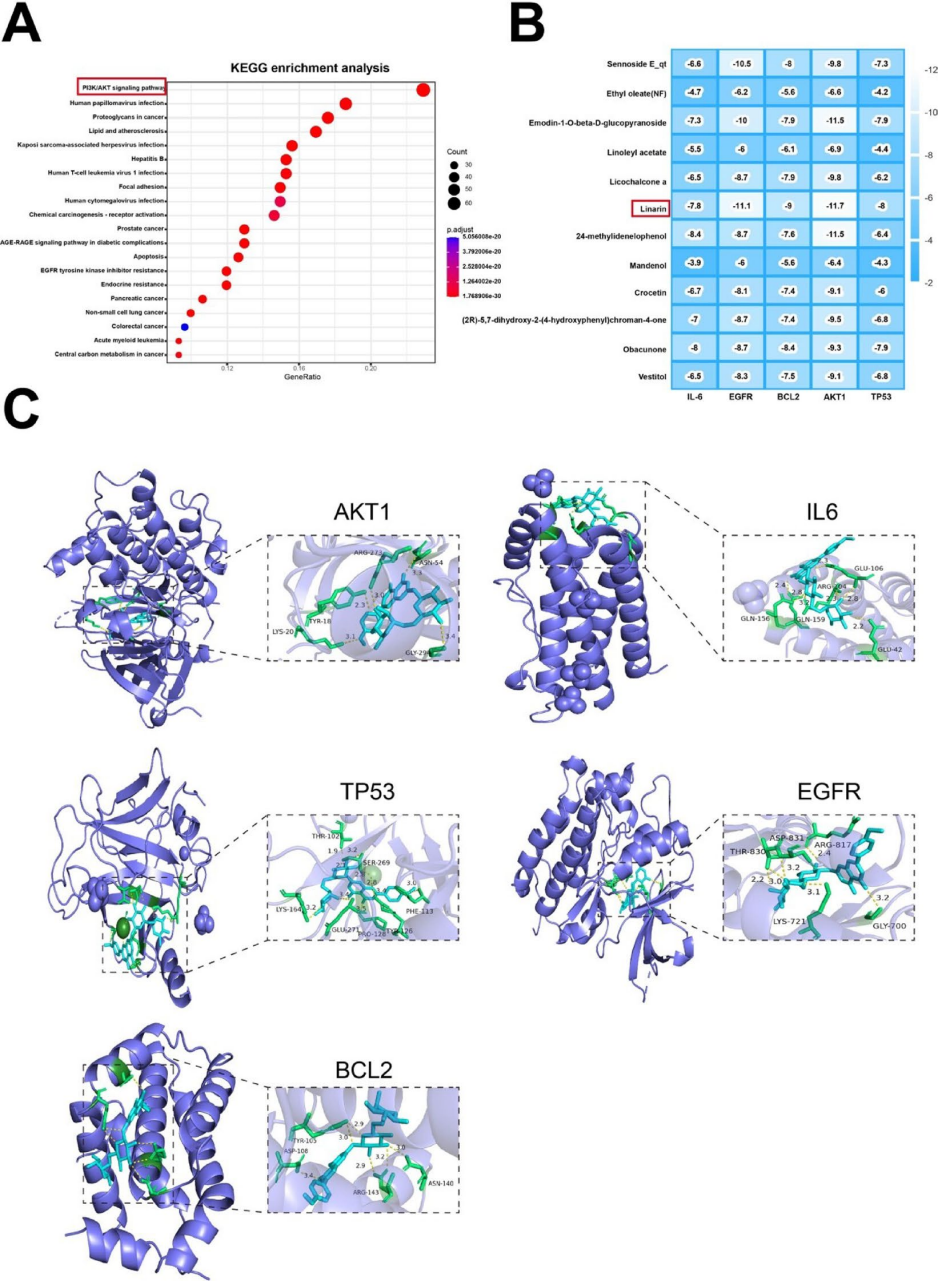
KEGG enrichment analysis and molecular docking results. (**A**) KEGG enrichment analysis. (**B**) Heatmap illustrating the binding affinity between core compounds and key targets as determined by molecular docking. (**C**) Molecular docking of linarin and 5 core targets

Based on the intersecting targets related to medicinal herbs for AP treatment, KEGG enrichment analysis was performed to assess representative pathways associated with these targets. The top 20 significantly enriched pathways are shown in Fig. 2A, with the PI3K/AKT pathway notably enriched. TP53, IL6, EGFR, BCL2, and AKT1 are overlapping targets between the top 11 core targets of the PPI network and relative targets in the PI3K/AKT pathway. To further investigate the direct interactions between the core compounds of medicinal herbs and key targets for AP treatment, molecular docking was carried out between 12 compounds and the 5 core targets. Lower binding energy indicates stronger binding affinity. The docking scores of the ligands and receptors are outlined in Fig. 2B. Linarin exhibited the strongest binding activity with 4 out of 5 core targets, among which its interaction with AKT1 was the most stable and had the highest binding affinity. The docking results of linarin are visualized in Fig. 2C.

Comprehensively analyzing the results above and relevant literature, we subsequently selected linarin and AKT1 for further investigation.

### Linarin treatment alleviated caerulein-induced AP mice

**Fig. 3:**
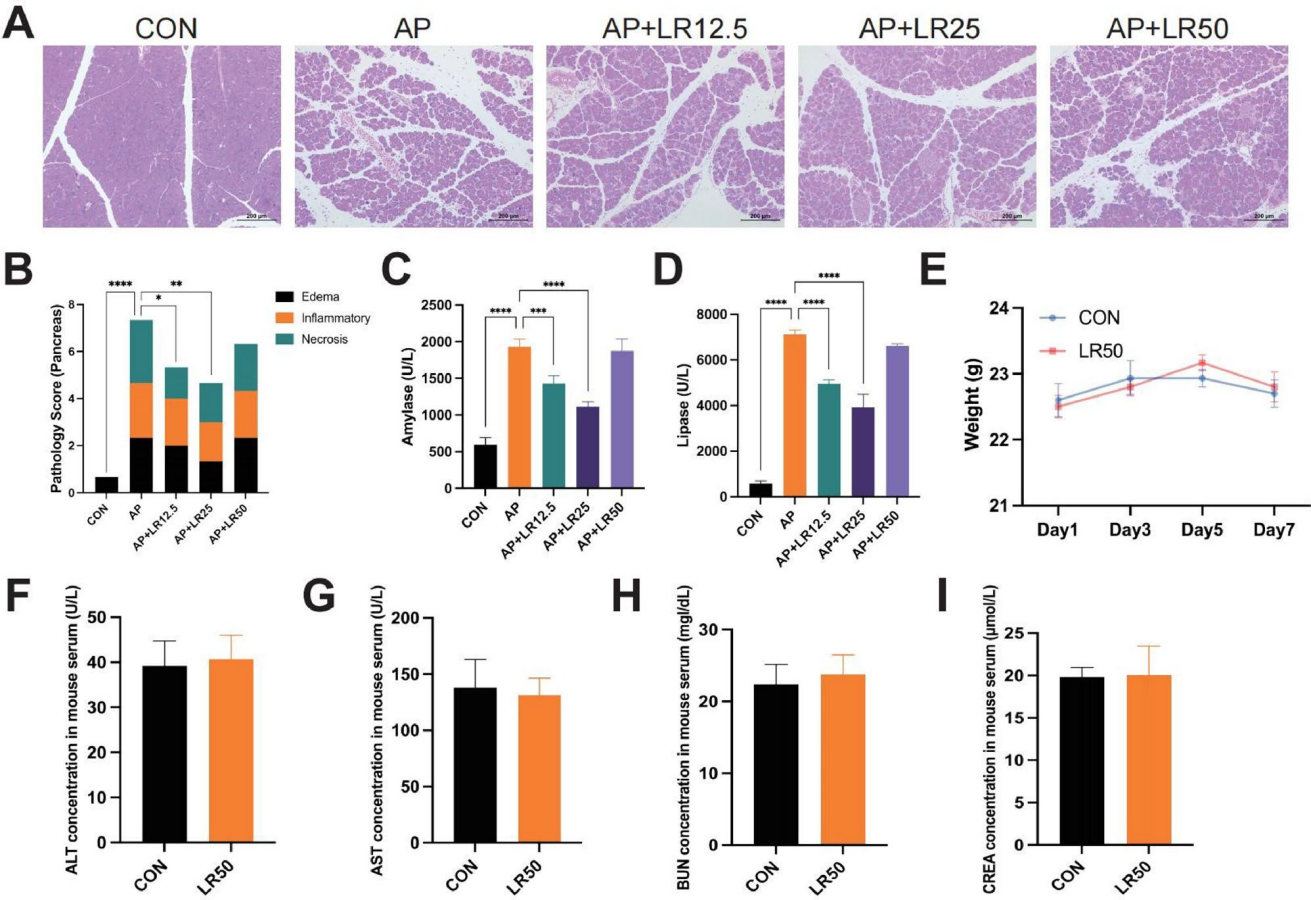
Effects of linarin on caerulein-induced AP mice and drug safety evaluation. (**A**) HE staining of pancreatic tissues. (**B**) Pancreatic tissue pathology score. (**C**) Serum amylase levels. (**D**) Serum lipase levels. (**E**-**I**) The dosage of 50 mg/kg linarin has no effect on mouse body weight, ALT, AST, BUN, and CREA levels. *n* = 5. *P < 0.05, **P < 0.01, ***P < 0.001, ****P < 0.0001 vs. AP group

Initially, we investigated the potential therapeutic effect of linarin in caerulein-induced AP mice. The results showed that the model group exhibited significant pancreatic edema, inflammatory cell infiltration, and necrotic alterations, with destroyed acinar cell structures. Additionally, amylase and lipase levels were significantly elevated compared to the CON group. However, following pretreatment with different concentrations of linarin, pancreatic tissue pathological damage was alleviated, and amylase and lipase levels were reduced to varying degrees, with the 25 mg/kg linarin showing the most effective efficacy (Fig. 3A-D).

Furthermore, the toxicity of linarin was evaluated, demonstrating that administration of 50 mg/kg linarin alone did not influence body weight (Fig. 3E). Also, levels of ALT, AST, BUN, and CREA showed no statistically significant differences when compared to the CON group (Fig. 3F-I). Collectively, the results above suggested that linarin could mitigate pancreatic injury to a certain extent, and 25 mg/kg linarin showed outstanding performance.

### Medium-dose linarin inhibited inflammation and apoptosis in AP mice

**Fig. 4:**
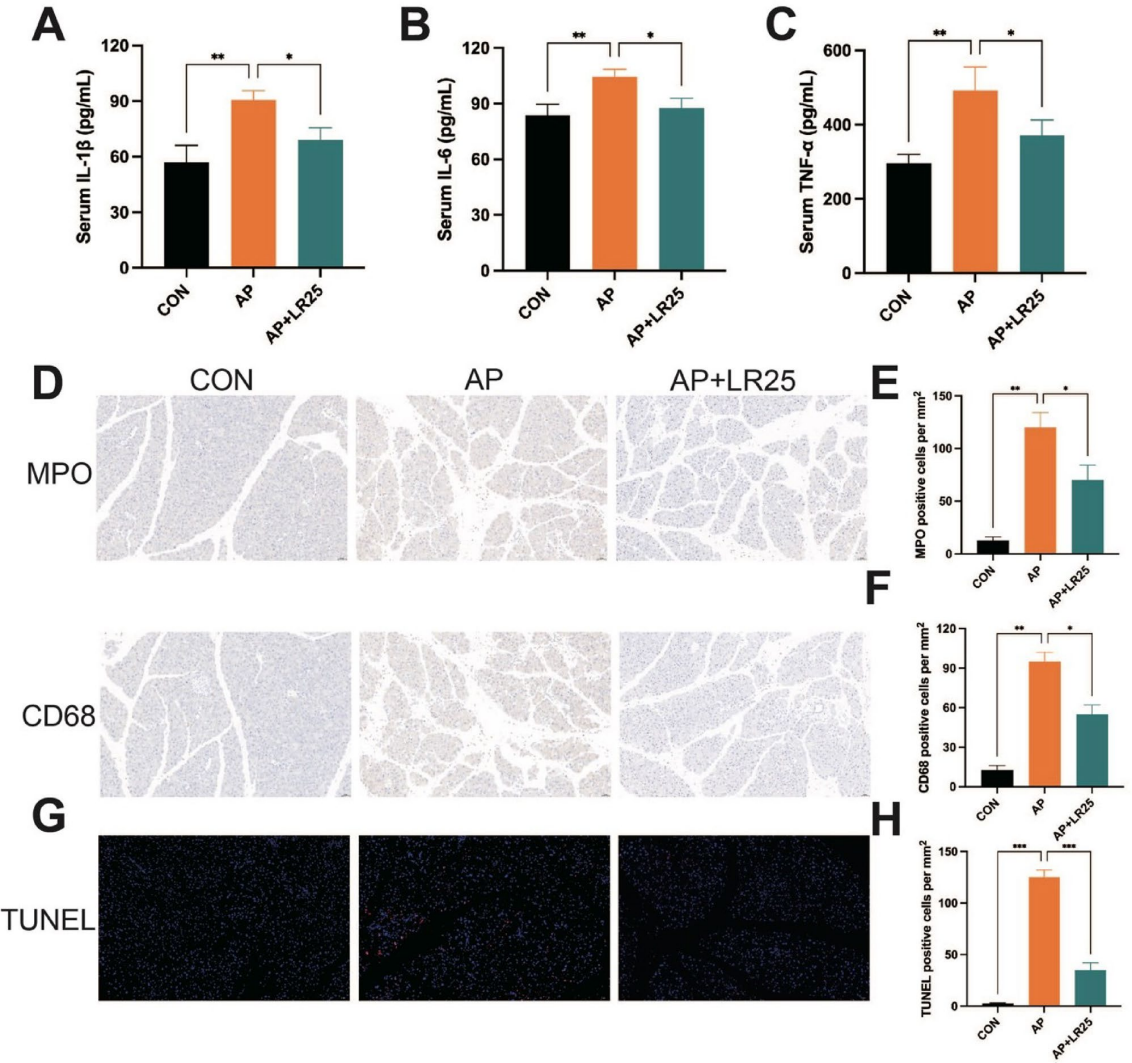
Effects of linarin on inflammation and apoptosis of caerulein-induced AP mice. (**A**) Bar graph of the levels of serum IL-1β. (**B**) Bar graph of the levels of serum IL-6. (**C**) Bar graph of the levels of serum TNF-α. (**D**) Immunohistochemical images of MPO and CD68 in the pancreatic tissues. (**E**) Statistical analysis of MPO expression. (**F**) Statistical analysis of CD68 expression. (**G**) TUNEL staining. (**H**) Statistical analysis of TUNEL-positive cells. *n* = 5. *P < 0.05, **P < 0.01, ***P < 0.001 vs. AP group

As shown in Fig. 4A-C, the serum levels of IL-1β, IL-6, and TNF-α in AP mice were significantly reduced after administration of 25 mg/kg linarin. Previous studies have reported that neutrophils and macrophages are closely associated with the severity of AP and play a critical role in systemic inflammatory responses and secondary organ damage in AP (Wan et al. 2020). MPO and CD68 are widely used as biomarkers for active neutrophils and macrophages, respectively (Yao et al. 2023). In this study, we assessed the expression of MPO and CD68 in pancreatic tissue by immunohistochemistry, which showed substantial recruitment in the AP group, whereas 25 mg/kg linarin treatment resulted in relatively fewer neutrophils and macrophages in the pancreatic tissue (Fig. 4D-F). Regarding acinar cell apoptosis is a critical contributor to the progression of AP, TUNEL staining was performed to evaluate apoptosis in pancreatic tissue. The results demonstrated that linarin significantly attenuated AP-induced pancreatic acinar cell apoptosis (Fig. 4G-H).

### Linarin attenuated caerulein-induced AP mice by modulating PI3K/AKT signaling cascade

**Fig. 5:**
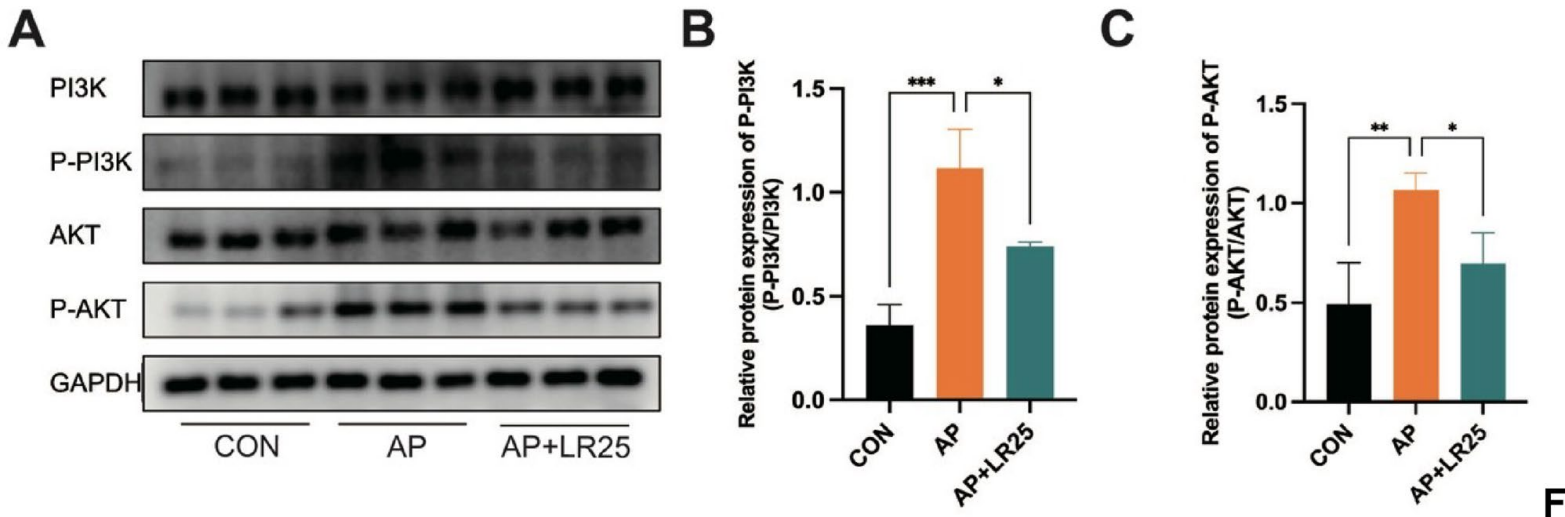
Linarin attenuated caerulein-induced AP mice via pathways involving PI3K/AKT signaling. (**A**) Western blots of PI3K/AKT signaling pathway. (**B**) Relative protein expression of P-PI3K. (**C**) Relative protein expression of P-AKT. *n* = 3. *P < 0.05, **P < 0.01, ***P < 0.001 vs. AP group

Based on network pharmacology and molecular docking results, the PI3K/AKT pathway is identified as the primary mechanism in the treatment of linarin on AP. To further validate this, we performed western blot experiments to assess the protein expression of key targets in the PI3K/AKT pathway. Compared with the CON group, the AP group showed significantly elevated levels of P-PI3K and P-AKT, which were restored following linarin treatment (Fig. 5).

### Effects of linarin on caerulein-induced AR42J cell injury

**Fig. 6:**
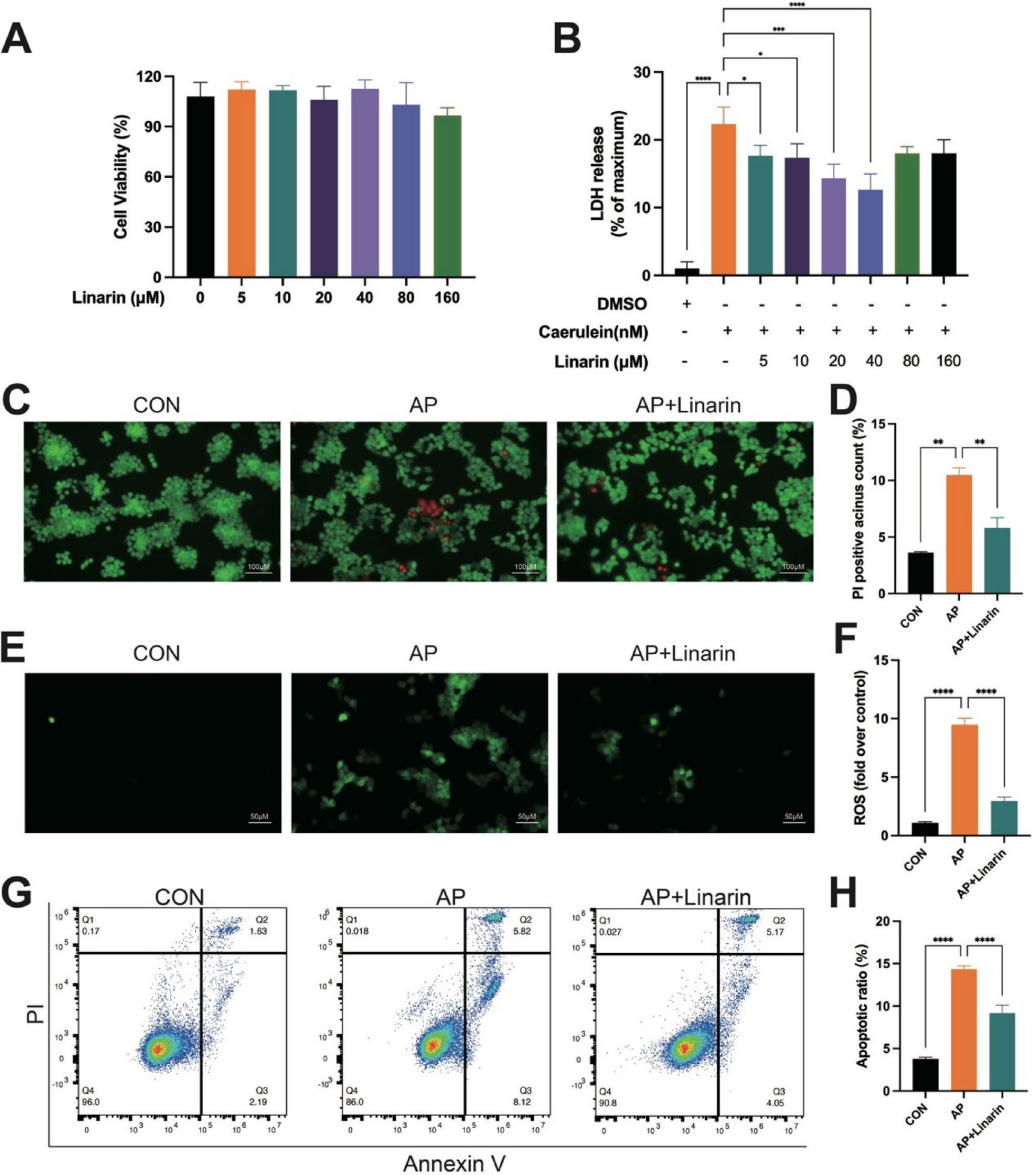
Effects of linarin on caerulein-induced AR42J cell. (**A**) CCK-8 assay of linarin on normal AR42J cells viability. (**B**) LDH release of linarin on caerulein-induced AR42J cells. (**C**) Calcein-AM/PI staining images. (**D**) Bar graph of PI-positive cells. (**E**-**F**) The ROS level in the AR42J cells. (**G**-**H**) The apoptosis level of the AR42J cells detected by flow cytometry. *n* = 5. *P < 0.05, **P < 0.01, ***P < 0.001, ****P < 0.0001 vs. AP group or DMSO group

To determine the optimal linarin concentration, CCK-8 assay was performed to assess its effect on AR42J cell viability, in which cells were exposed to varying concentrations of linarin for 24 h. The results showed that 160 µM linarin displayed no observable pharmacotoxicity on normal AR42J cells, indicating its safety for further experiments (Fig. 6A). Next, the pharmacological effect of linarin on AR42J cell necrosis was evaluated using a caerulein-induced cell injury model, which demonstrated that linarin significantly inhibited LDH release, with the best efficacy observed at 40 µM (Fig. 6B). Furthermore, 40 µM linarin treatment significantly reduced the proportion of PI-positive cells in caerulein-induced AR42J cells, as shown in Fig. 6C-D. Based on these findings, 40 µM was selected as the optimal concentration for subsequent experiments.

### Linarin reduced ROS generation and apoptosis in AR42J cells

ROS plays a crucial role in initiating inflammation in pancreatic acinar cells and is closely associated with the pathogenesis of AP. Previous studies have reported that ROS can directly oxidize biomolecules in the pancreatic acinar cell membrane, including lipids, proteins, and nucleic acids, leading to membrane degradation and necrosis. Additionally, ROS acts as a secondary messenger in intracellular signaling, promoting pro-inflammatory cascades (Ahn et al. 2020). To further investigate the effect of linarin on ROS levels in caerulein-treated AR42J cells, DCFH-DA staining was performed. The results showed that linarin pretreatment effectively diminished ROS production (Fig. 6E-F).

The effect of linarin on apoptosis in caerulein-exposed AR42J cells was evaluated using flow cytometry. The results demonstrated that, compared to the CON group, the AP group exhibited a higher proportion of apoptotic cells, whereas linarin treatment significantly decreased apoptosis in caerulein-treated AR42J cells (Fig. 6G-H).

### Effect of linarin on PI3K/AKT signaling in caerulein-induced AR42J cells

**Fig. 7:**
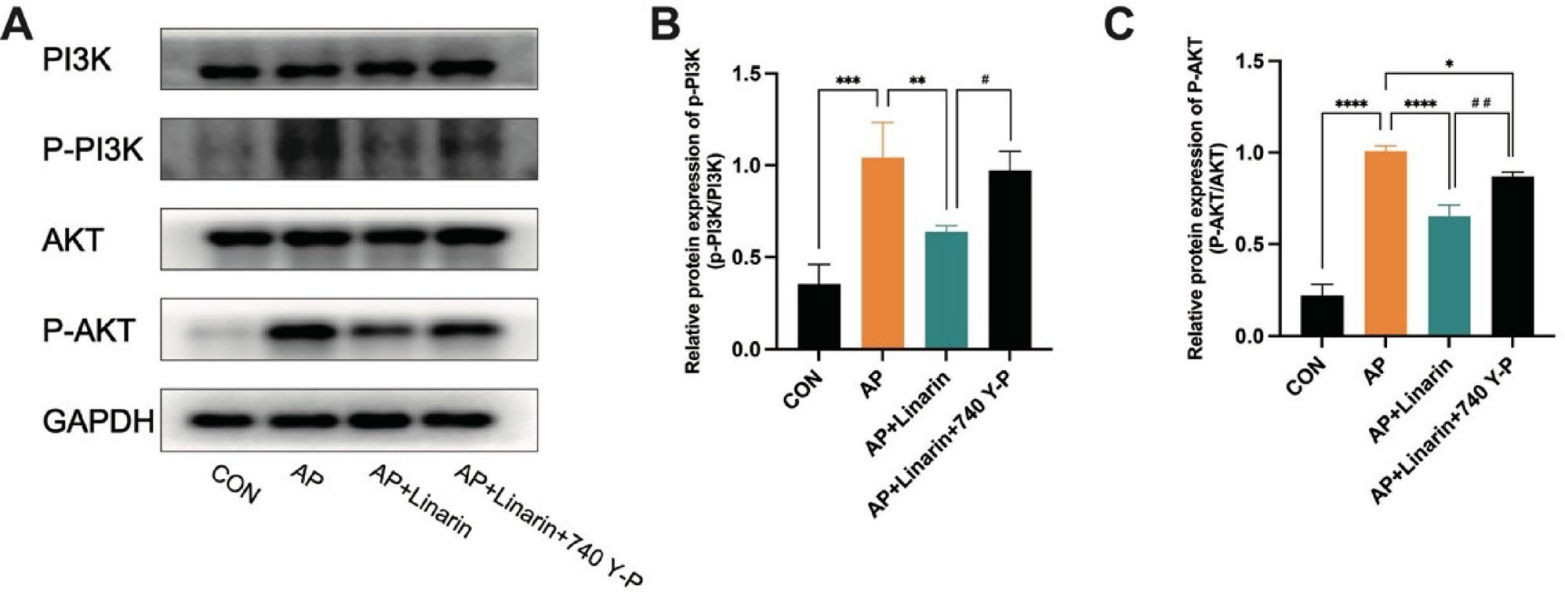
Effect of linarin on PI3K/AKT signaling in caerulein-induced AR42J cells. (**A**) Western blots of PI3K/AKT signaling pathway. (**B**) Relative protein expression of P-PI3K. (**C**) Relative protein expression of P-AKT. *n* = 3. *P < 0.05, **P < 0.01, ***P < 0.001, ****P < 0.0001 vs. AP group. ^#^*P* < 0.05, ^##^*P* < 0.01 vs. AP + Linarin group

Next, we further explored the specific mechanism by which linarin exerted its protective effects through the regulation of the PI3K/AKT signaling pathway. Consistent with the in vivo experiments, as shown in Fig. 7, the expression levels of P-PI3K and P-AKT were significantly higher in caerulein-induced AR42J cells than in the CON group. However, 40 µM linarin markedly downregulated the expression of these phosphorylated proteins. This inhibitory effect was reversed by the PI3K/AKT agonist 740 Y-P, which restored P-PI3K and P-AKT levels upon its pretreatment. These findings confirmed that linarin inhibited the PI3K/AKT signaling pathway in caerulein-induced AR42J cells.

## Discussion

AP is an inflammatory pancreatic disease, characterized primarily by abdominal pain and elevated levels of pancreatic enzymes in the blood (Lee and Papachristou 2019). Despite extensive research, the underlying mechanisms driving the onset and progression of AP remain unclear, and current treatments primarily focus on symptom relief, such as inhibiting pancreatic enzyme secretion or alleviating severe pain (Jiang et al. 2020). Modern studies have demonstrated promising therapeutic effects of TCMs in AP treatment, however, their specific mechanisms demand to be explored (Han et al. 2024; Wang et al. 2024a). Network pharmacology, an emerging technique known for its systematic and integrative features, provides a promising novel method for studying the complex TCM system (Li et al. 2023b). It is noted that previous studies have primarily concentrated on the efficacy and mechanisms of individual herbs or decoctions. Considering the construction of compound-target networks in network pharmacology and herbal medicines’ multi-component, multi-target characteristics, we comprehensively analyzed medicinal herbs documented for AP treatment to identify their common core active compounds and targets. This strategy has been successfully applied to other diseases but has yet to be reported in the context of AP (Yu et al. 2024).

Initially, we screened 62 common compounds from 37 medicinal herbs and identified core active compounds and targets through network topology analysis. Five target genes (TP53, IL6, EGFR, BCL2, and AKT1) were enriched in the most significant signaling pathway, the PI3K/AKT signaling cascade. Molecular docking analyses were then conducted to evaluate the binding affinity between these core active compounds and the five target genes. The results revealed that linarin exhibited the strongest binding affinity to the vast majority of core targets. Furthermore, in vivo and in vitro experiments confirmed that linarin alleviated pancreatic damage and inflammatory cell infiltration in AP and inhibited the activation of the PI3K/AKT signaling pathway. Based on these findings, we successfully employed this novel effectiveness-directed target-discovery network pharmacology strategy to identify and validate the potential therapeutic effect of linarin on AP, among which the PI3K/AKT signaling pathway, particularly the AKT1 target plays a pivotal role.

Linarin, a glycosylated flavonoid, enhances its biological properties by reducing toxicity and increasing bioavailability when compared to other non-glycosylated flavonoids (Slamova et al. 2018). This compound has been extensively investigated for its diverse pharmacological activities, with accumulating evidence supporting its dual potential as both a therapeutic agent and dietary supplement. To date, linarin has been identified in several medicinal plants, including C. japonicum, C. indicum, and Artemisia capillaris Thunb (Mottaghipisheh et al. 2021). Feng et al. investigated the pharmacokinetics of linarin after oral administration and found that linarin was rapidly and extensively distributed across various tissues within one minute, with the pancreatic tissue maintaining a relatively high concentration after 8 h (Feng et al. 2016). This suggests that linarin can pass through the blood-pancreas barrier (BPB), which is a crucial factor hindering drug delivery to the inflammatory pancreas (Jiang et al. 2020). Moreover, linarin has demonstrated anti-inflammatory pharmacological activity in various inflammatory diseases. In obese mouse models, linarin effectively ameliorates insulin resistance and inflammatory responses through modulation of the c-FOS/ARG2 signaling axis (Liu et al. 2025). In acute liver injury models, linarin prevents hepatic damage by attenuating oxidative stress, suppressing TLR4/MyD88 and JNK/p38/ERK-mediated inflammatory pathways, while enhancing Beclin 1/LC3II-regulated autophagic flux (Li et al. 2023a). Additionally, linarin also exhibits promising therapeutic effects in DSS-induced colitis models, with proposed mechanisms involving intestinal barrier restoration, anti-inflammatory actions, and gut microbiota modulation (Jin et al. 2022). Although there is no direct study on its effects in AP, previous research has shown that linarin is a potent inhibitor of α-amylase and lipase (Chenafa et al. 2022; Kim et al. 2016). Taken together, these findings imply that linarin may be an excellent candidate for AP treatment. Subsequently, our network pharmacology analysis, in vivo, and in vitro experiments successfully validated the therapeutic potential of linarin in AP.

AKT is a multifunctional kinase associated with a wide range of cellular functions, including metabolism, survival, migration, and gene expression in most cell lineages. Also, it is well known that AKT plays a central role in both physiological and pathological signal transduction (Siddika et al. 2022). In mammals, the AKT protein family is generally divided into three isoforms: AKT1, AKT2, and AKT3, which are products of different genes (Karege et al. 2010). AKT1 has been found to be crucial for acute inflammation, significantly reducing edema formation as well as neutrophil and monocyte infiltration (Di Lorenzo et al. 2009). Studies have shown that the expression of all three AKT isoforms is significantly elevated during AP (Chen et al. 2020). Additionally, several literature have reported that inhibition of the PI3K/AKT pathway exhibits promising effects in downregulating inflammation in AP animal models (Luo et al. 2020; Xu et al. 2024b). However, the application of AKT1 inhibitors in AP has become a controversial topic in recent years, mainly due to concerns regarding the impairment of acinar regeneration and the promotion of acinar-to-ductal metaplasia (Sarker and Steiger 2020). In our study, we also identified AKT1 as an important therapeutic target for AP, and the protective effect of linarin in AP was found to be dependent on the PI3K/AKT pathway through pretreatment with agonist 740 Y-P. Based on the aforementioned findings and Chen et al.‘s dialectical description of AKT inhibitors (Chen et al. 2020), the safety profile of linarin as an AKT inhibitor targeting the PI3K/AKT pathway requires further evaluation. While current data on long-term linarin administration remain limited, existing studies have demonstrated its broad pharmacological activities. Notably, Oh et al. reported no observable acute or chronic toxicity in mice following eight-week treatment with linarin at 20 mg/kg (Oh et al. 2023), suggesting its favorable safety profile for potential clinical applications. However, comprehensive toxicity assessments remain warranted, including extended chronic toxicity studies, detailed organ-specific toxicity evaluations, and comparative analyses with classical AKT inhibitors (e.g., MK-2206) regarding their effects on acinar cell death and proliferation. These investigations would be crucial for precisely defining linarin’s therapeutic window and pathway specificity, thereby facilitating its clinical translation.

Although this study screened core active compounds and targets for AP through network pharmacology and validated the therapeutic effect of linarin on AP as well as the involvement of the PI3K/AKT pathway through in vivo and in vitro experiments, there are still certain limitations. First, the effects of other active compounds and targets identified by network pharmacology were not experimentally validated. Second, the selection of important nodes in the network was based solely on degree centrality. Incorporating additional network metrics like betweenness and closeness centrality would provide a more comprehensive assessment of strategic connectivity within the network. Additionally, the specific role of AKT1 in AP remains underexplored.

## Conclusion

This study utilized a novel network pharmacology approach to summarize the common mechanisms and targets of medicinal plants used in AP treatment. We identified AKT1 as a core therapeutic target and linarin as a promising candidate for AP treatment in the preclinical stage. These findings provide a new perspective and direction for AP therapeutic exploration, though further toxicological and clinical studies are warranted.

## Supplementary Information

Below is the link to the electronic supplementary material.

Supplementary Material 1

Supplementary Material 2

Supplementary Material 3

Supplementary Material 4

Supplementary Material 5

Supplementary Material 6 Supplementary Material 6

## Data Availability

Data will be made available on request.

## Abbreviations

AP: Acute pancreatitis
SAP: Severe acute pancreatitis
TCM: Traditional Chinese medicine
DL: Drug-likeness
OB: Oral bioavailability
LR: Linarin
DMSO: Dimethyl sulfoxide
HE: Hematoxylin and eosin
CCK-8: Cell Counting Kit-8
Calcein-AM: Calcein Acetoxymethylester
PI: Propidium Iodide
ROS: Reactive Oxygen Species
SD: Standard deviation
IQR: Interquartile range
ANOVA: One-way analysis of variance
BPB: Blood-pancreas barrier
