# Supplementary material for: Unveiling the common mechanisms and therapeutic targets of medicinal herbs in acute pancreatitis: a network pharmacology and experimental validation approach
Source: Bioresour Bioprocess. 2025 Jul 30;12(1):82. doi: 10.1186/s40643-025-00925-1 (PMC12311076; doi:10.1186/s40643-025-00925-1)

**Unveiling the Common Mechanisms and Therapeutic Targets of Medicinal herbs in Acute Pancreatitis: A Network Pharmacology and Experimental Validation Approach**

Yuxin Shi^12^*, Yan Jia^12^*, Ya Liu^12^, Hanyue Wang^12^, Honghui Liu^12^, Yilin Huang^12^, Peiyan Chen^12^, Jie Peng^12#^

^1^Department of Gastroenterology, Xiangya Hospital, Central South University, Changsha, Hunan 410008, China; ^2^National Clinical Research Center for Geriatric Disorders, Xiangya Hospital, Central South University, Changsha, Hunan 410008, China.

Yuxin Shi^12*^

Yan Jia^12*^

*These authors contributed equally to this work

Correspondence: Jie Peng

Department of Gastroenterology, Xiangya Hospital, Central South University; National Clinical Research Center for Geriatric Disorders, Xiangya Hospital, Central South University

Email: pengjie2014@csu.edu.cn

**Original Western blot image**

**Tips:**

1. A Prestained Protein Marker (Cat# P8028L, UElandy Inc., China) was utilized as a molecular weight reference in western blot experiments. The molecular weights of the marker bands, as specified in the instructions, are shown in the figure below.


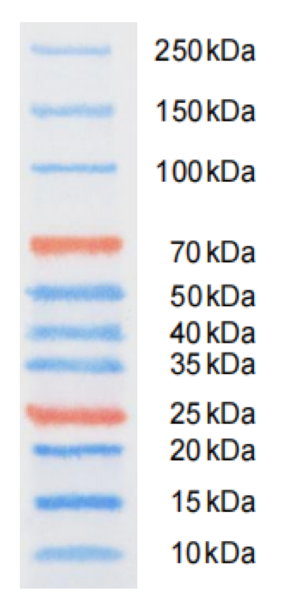


1. During primary antibody incubation, the phosphorylated antibody and internal control antibody were incubated first, followed by elution using the stripping buffer (Cat# ES-8712, ECOTOP SCIENTIFIC, China). Subsequently, the non-phosphorylated antibody was incubated in sequence.
2. The bands in the raw data are indicated by red arrows.

Figure 5: P-AKT/AKT/GAPDH


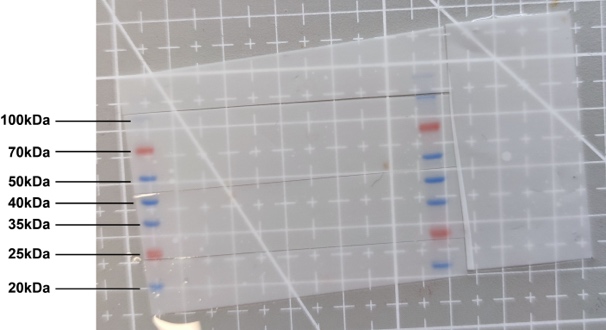


P-AKT: 60kDa (Abmart, China, diluted 1:1000)


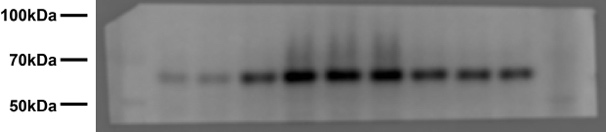

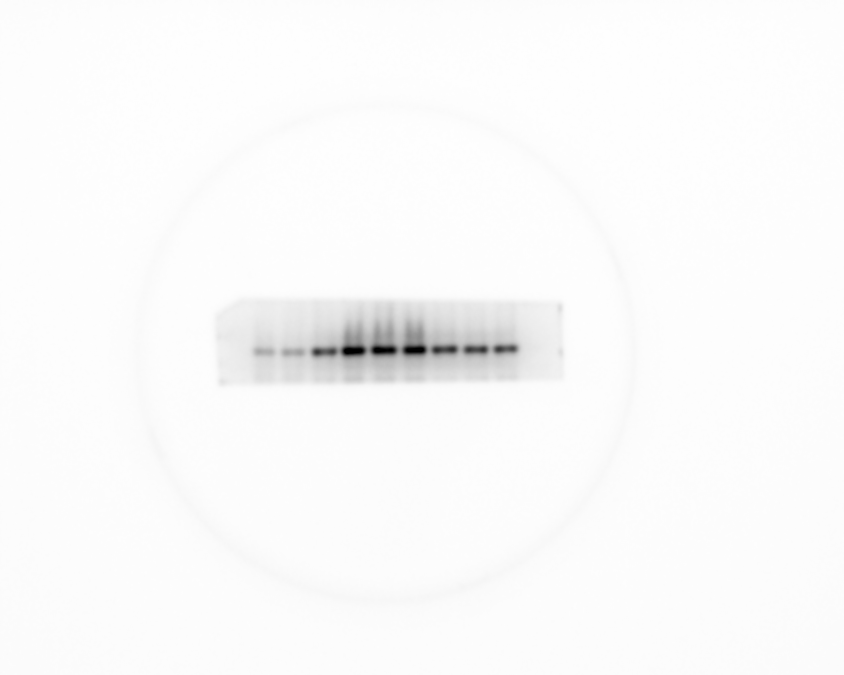


AKT: 60kDa (Abmart, China, diluted 1:1000)


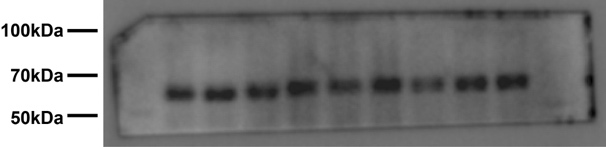

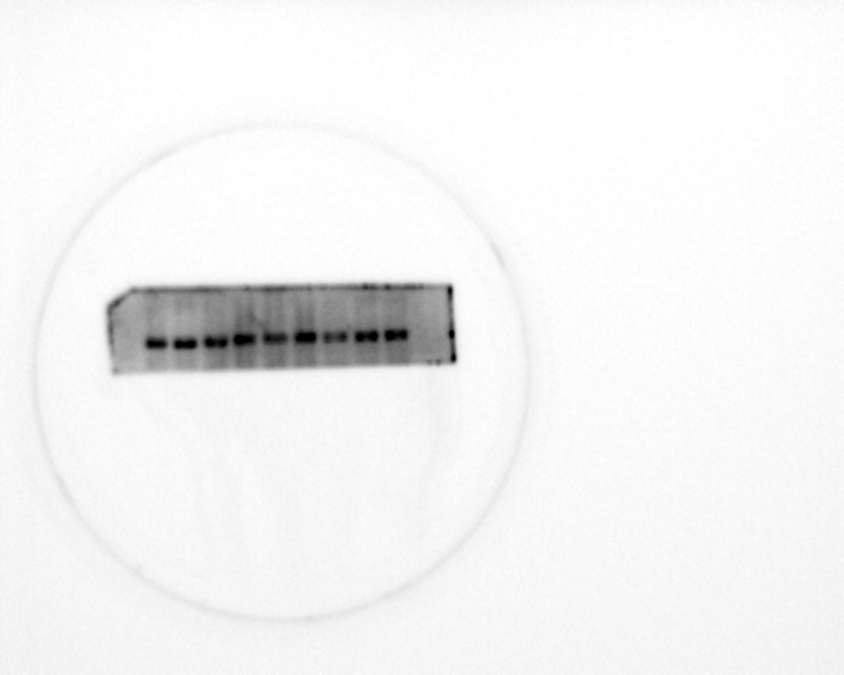


GAPDH: 36kDa (Beyotime, China, diluted 1:2500)


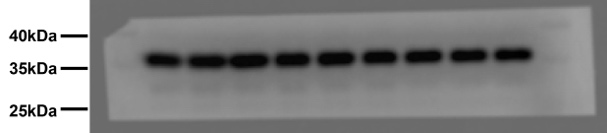

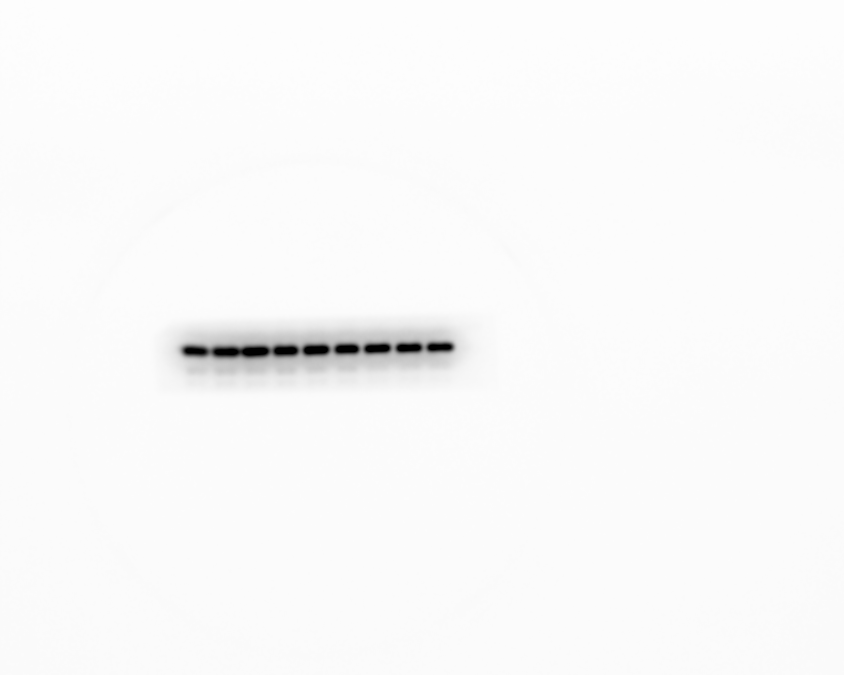


Figure 5: P-PI3K/PI3K/GAPDH


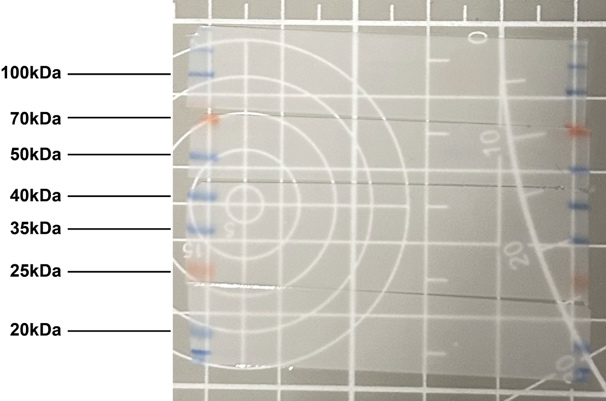


P-PI3K: 85kDa (Cell Signaling Technology, USA, diluted 1:1000 )


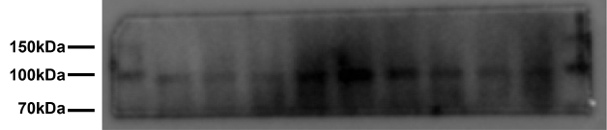

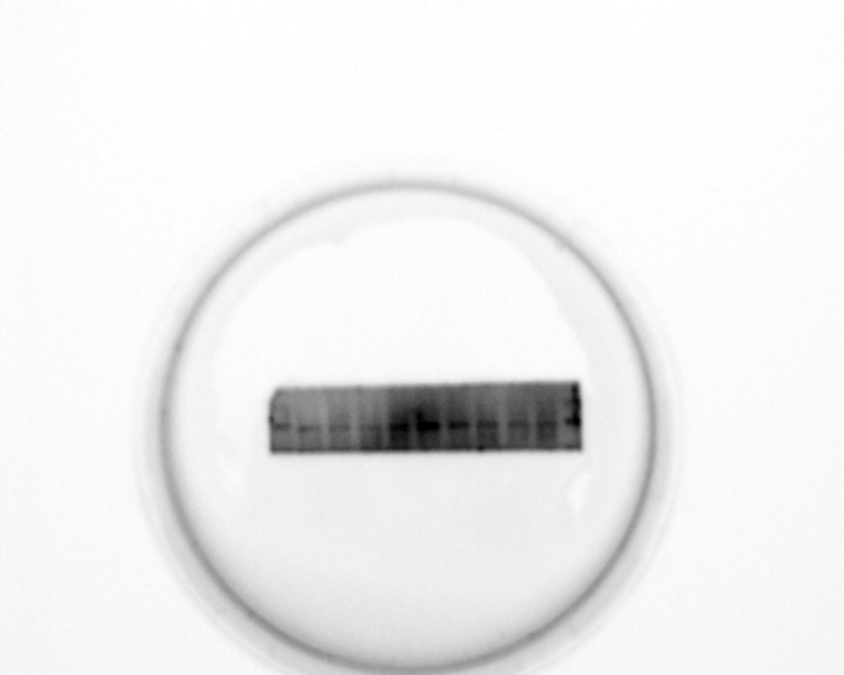


PI3K: 85kDa (Abmart, China, diluted 1:1000)


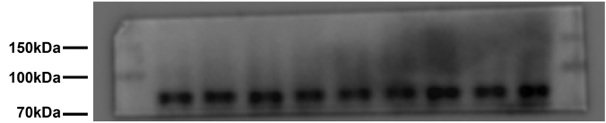

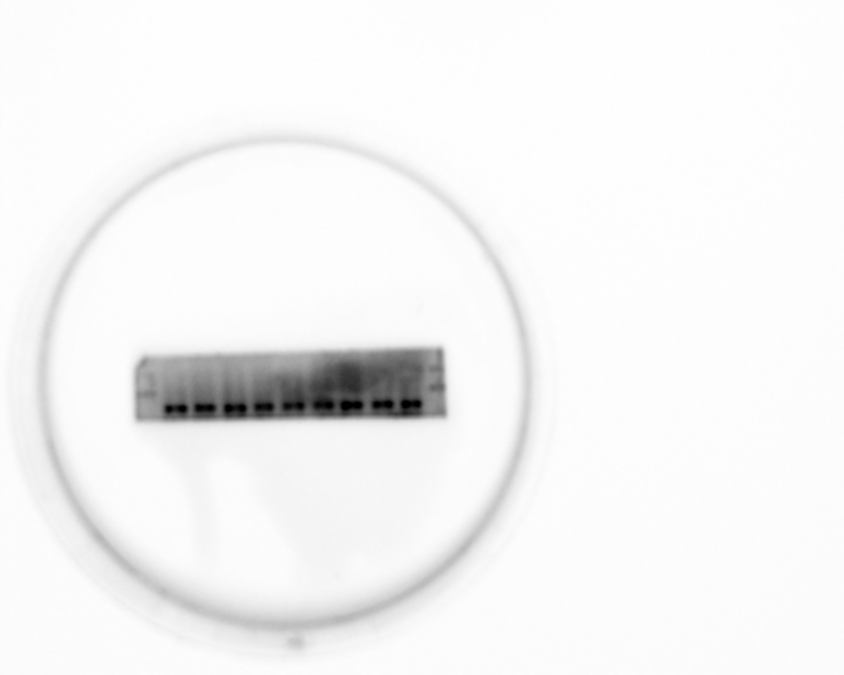


GAPDH: 36kDa (Beyotime, China, diluted 1:2500)


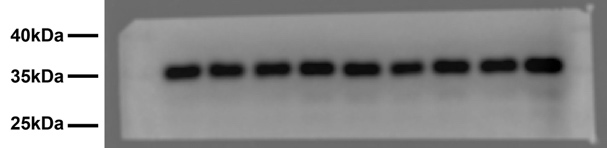

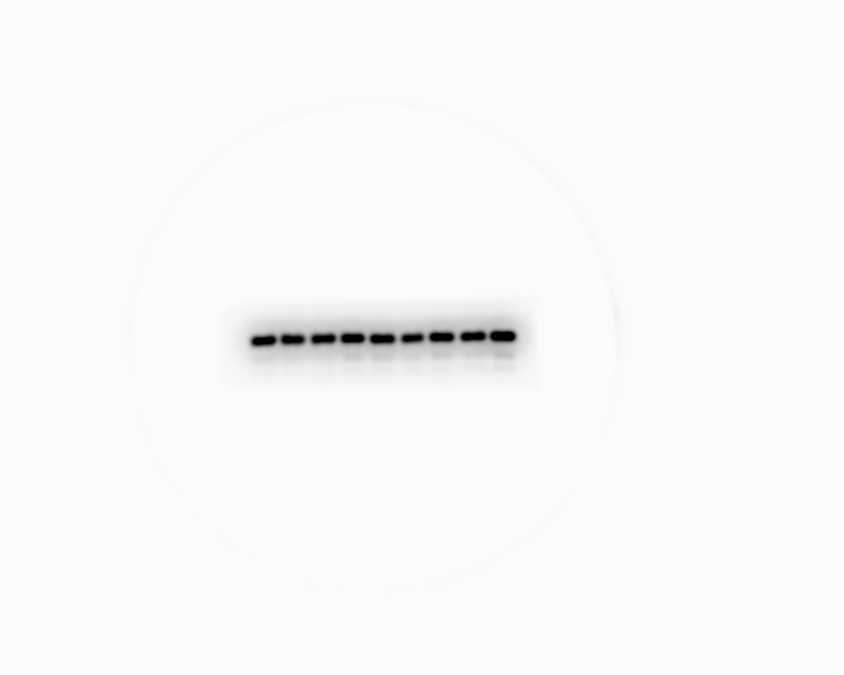


Figure 7: P-PI3K/PI3K/P-AKT/AKT/GAPDH

Repeat 1


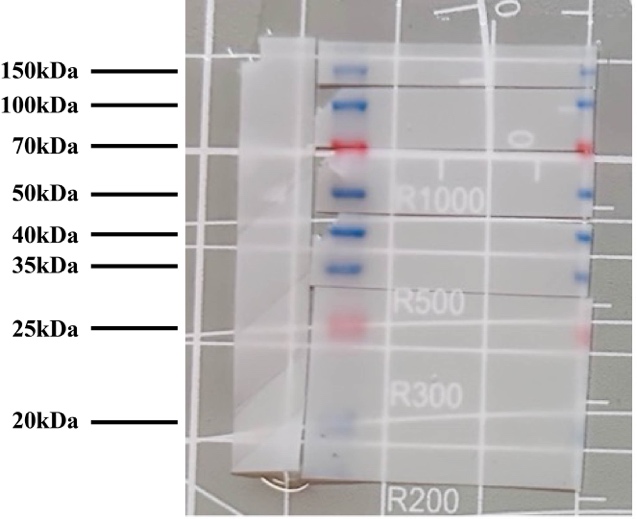


P-PI3K: 85kDa


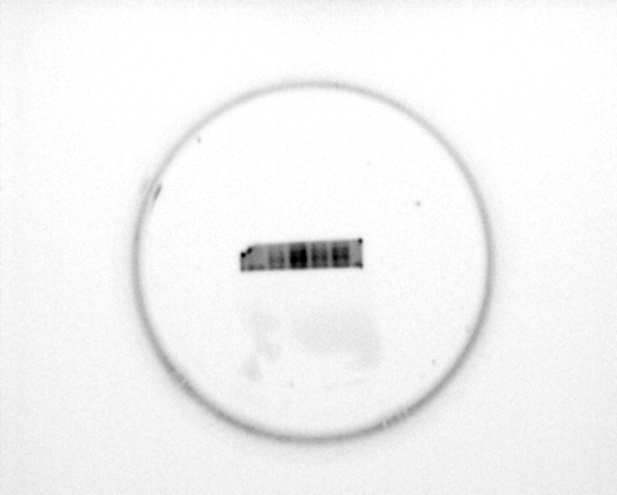


PI3K: 85kDa


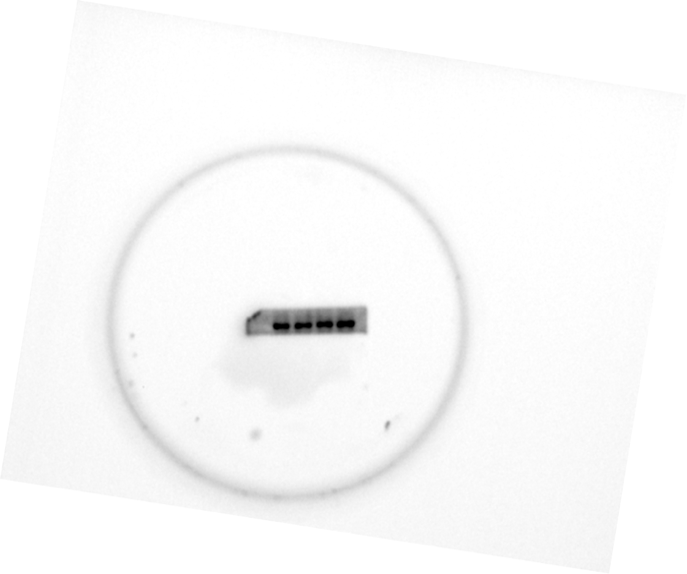


P-AKT: 60kDa


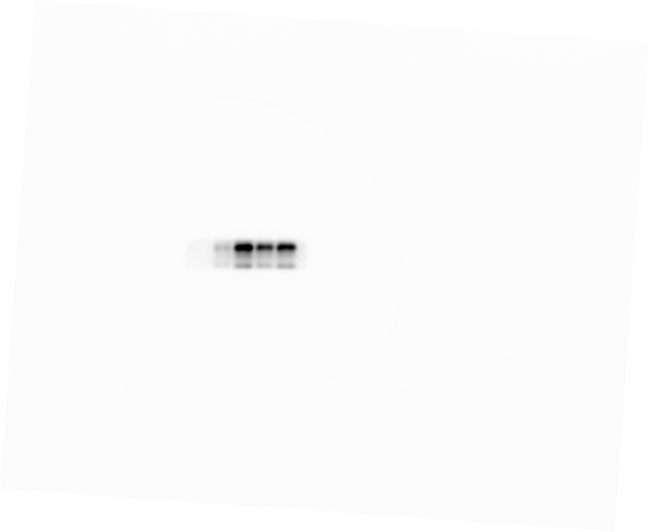


AKT: 60kDa


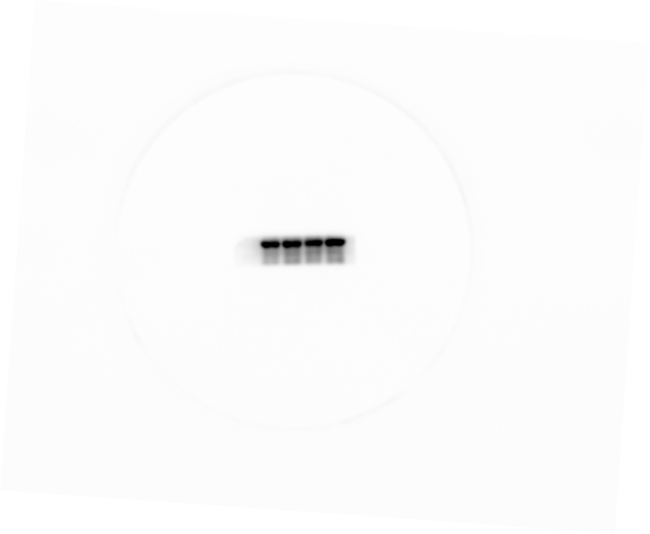


GAPDH: 36kDa


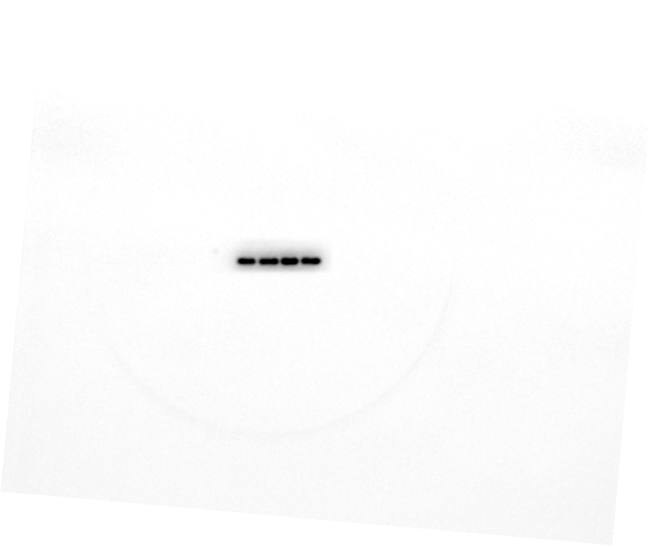


Repeat 2


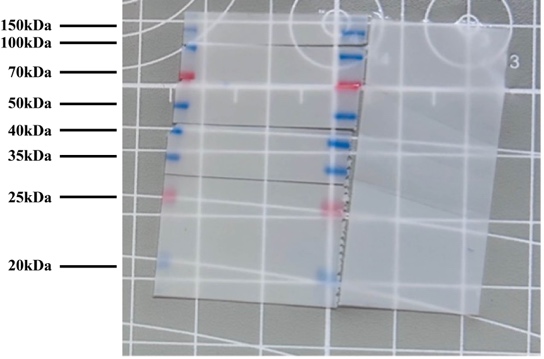


P-PI3K: 85kDa


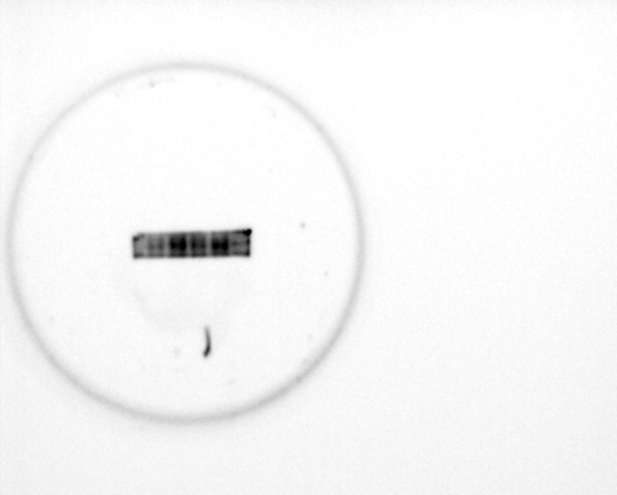


PI3K: 85kDa


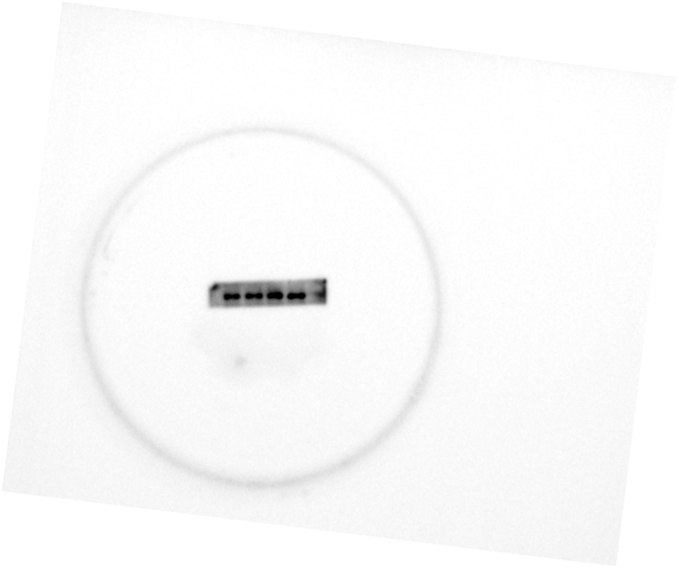


P-AKT: 60kDa


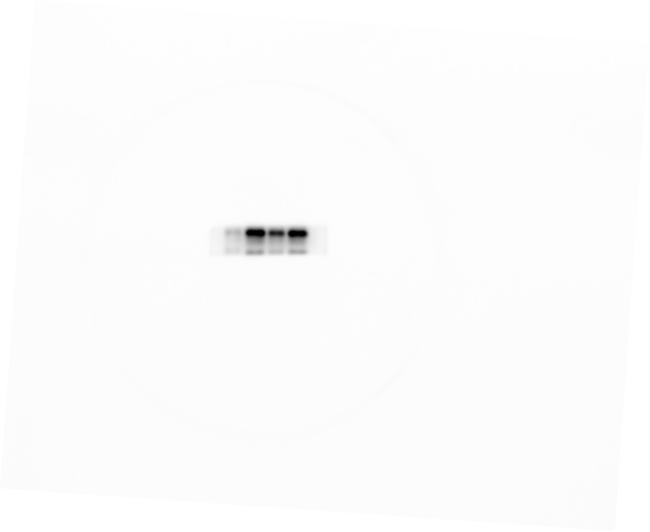


AKT: 60kDa


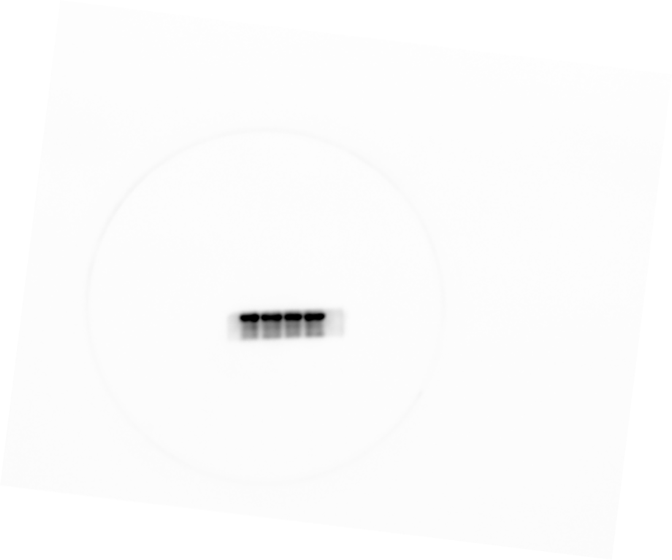


GAPDH: 36kDa


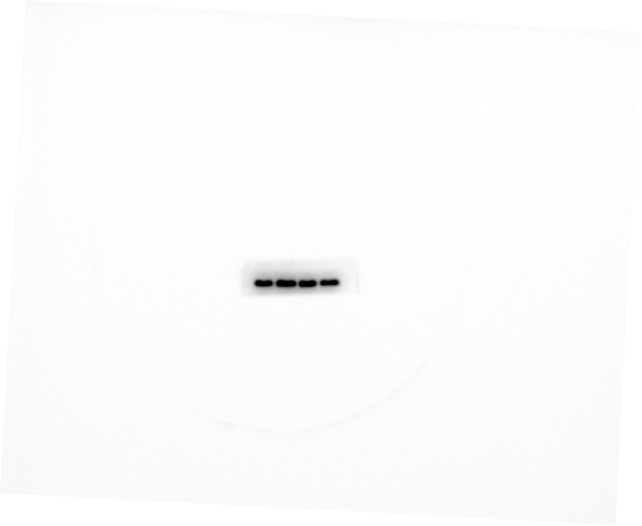


Repeat 3


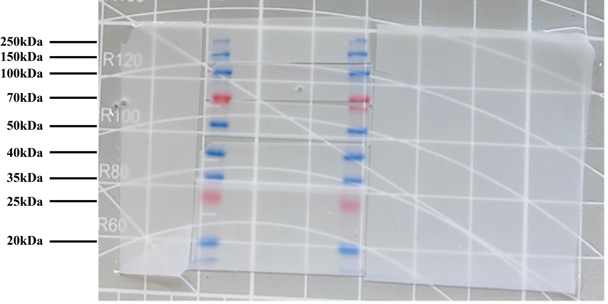


P-PI3K: 85kDa


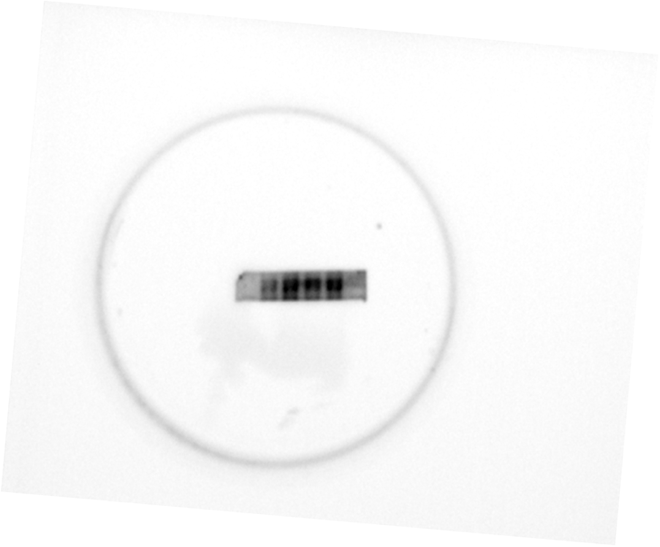


PI3K: 85kDa


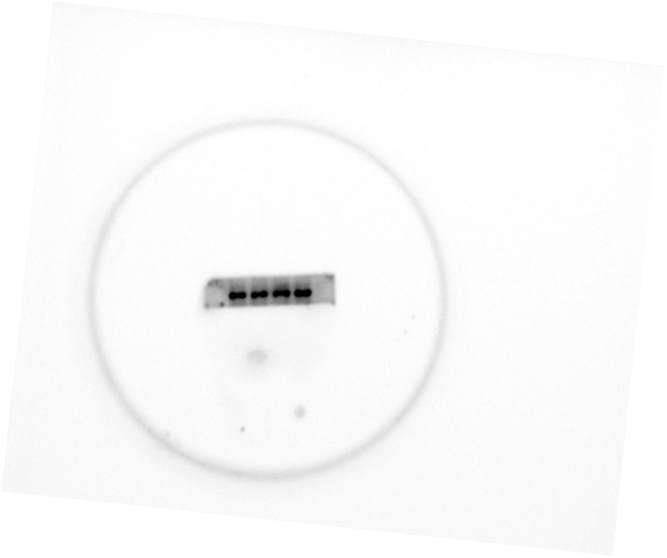


P-AKT: 60kDa


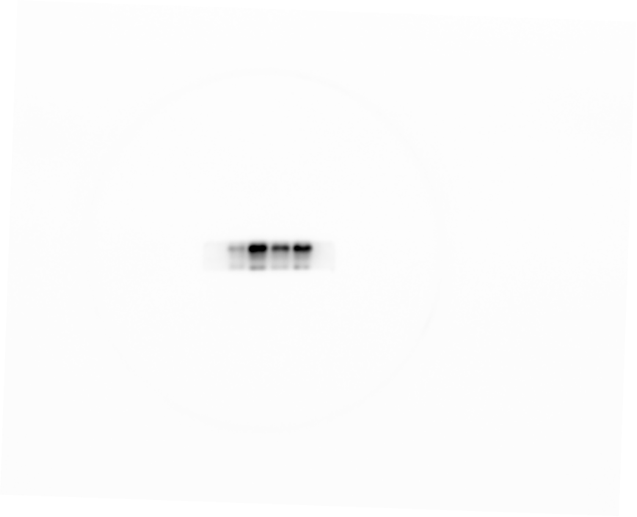


AKT: 60kDa


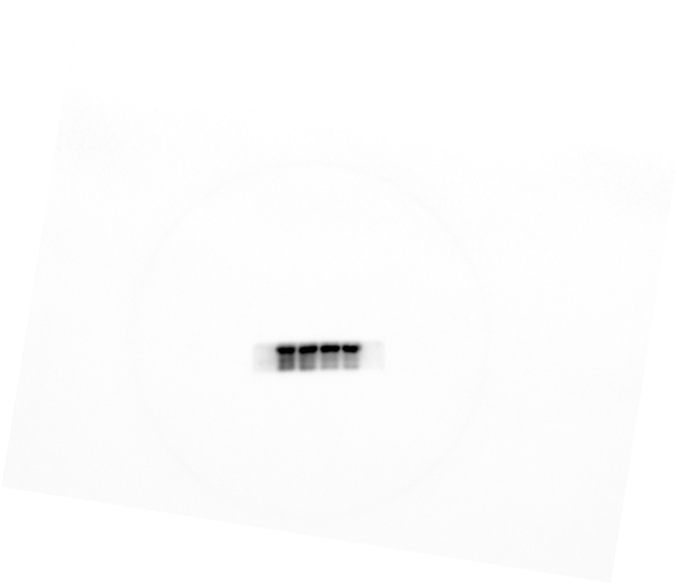


GAPDH: 36kDa


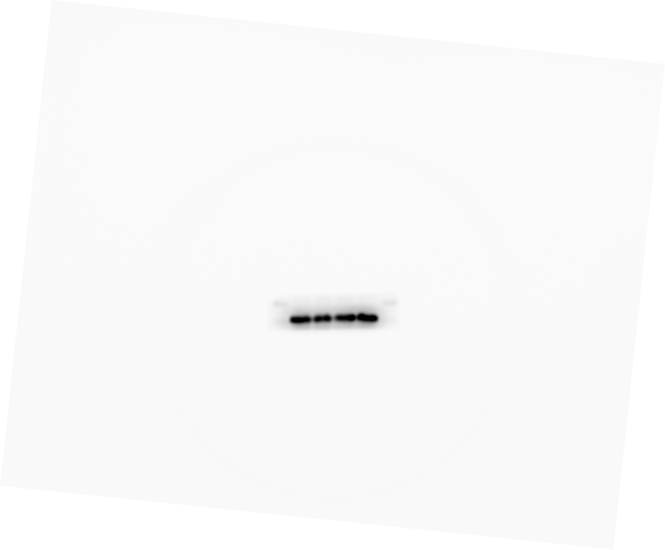

Supplement: Supplementary file 4 — Supplementary Material 4 [file 40643_2025_925_MOESM4_ESM.docx]
