# Supplementary figures and images for: Unveiling the common mechanisms and therapeutic targets of medicinal herbs in acute pancreatitis: a network pharmacology and experimental validation approach
Source: Bioresour Bioprocess. 2025 Jul 30;12(1):82. doi: 10.1186/s40643-025-00925-1 (PMC12311076; doi:10.1186/s40643-025-00925-1)

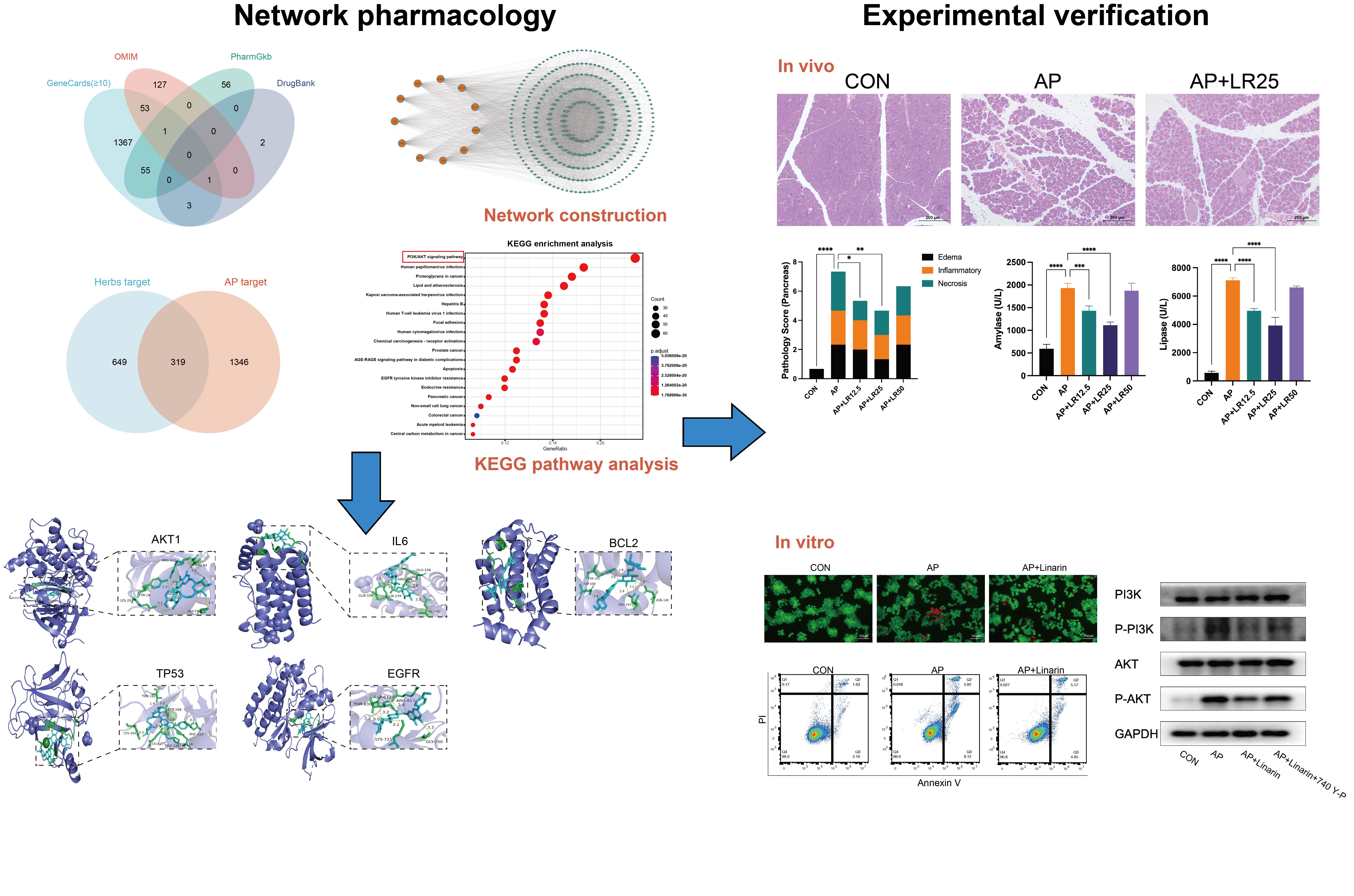

Supplement: Supplementary file 6 — Supplementary Material 6 [file 40643_2025_925_MOESM6_ESM.jpg]
