# Supplementary material for: Unveiling the common mechanisms and therapeutic targets of medicinal herbs in acute pancreatitis: a network pharmacology and experimental validation approach
Source: Bioresour Bioprocess. 2025 Jul 30;12(1):82. doi: 10.1186/s40643-025-00925-1 (PMC12311076; doi:10.1186/s40643-025-00925-1)
Supplement: Supplementary file 5 — Supplementary Material 5 [file 40643_2025_925_MOESM5_ESM.pdf]

Dear editors and reviewers,

We sincerely appreciate the continued interest in our findings and the additional constructive comments from the editors and reviewers on our manuscript entitled "Unveiling the Common Mechanisms and Therapeutic Targets of Medicinal Herbs in Acute Pancreatitis: A Network Pharmacology and Experimental Validation Approach." We have carefully addressed all the new points raised in the second round of review, implementing the suggested revisions as detailed below in blue. All changes in the revised manuscript have been highlighted for easy reference.

We are truly grateful for the opportunity to further refine our manuscript and hope that the revised version aligns with the journal's standards. Any additional feedback would be greatly appreciated.

Response to the comments of the Editor

Your manuscript 'Unveiling the Common Mechanisms and Therapeutic Targets of Medicinal herbs in Acute Pancreatitis: A Network Pharmacology and Experimental Validation Approach' (BIOB-D-25-00148R1) has been assessed by our reviewers. Based on these reports, and my own assessment as Editor, I am pleased to inform you that it is potentially acceptable for publication in Bioresources and Bioprocessing, once you have carried out some essential revisions suggested by our reviewers.

Thank you for your email and the positive assessment of our manuscript. We sincerely appreciate the reviewers' constructive comments and the opportunity to further improve our work. We have carefully addressed all the essential revisions suggested by the reviewers. The point-by-point responses are detailed in blue, and all corresponding changes in the manuscript have been highlighted. We hope the revised version meets the journal's requirements. Please let us know if any additional modifications are needed. Thank you again for your time and consideration.

Response to the comments of Reviewer 1

The article seems good to me. In the discussion section instead of mentioning the future prospects for your study you can also cite literature on efficacy of linarin in other similar conditions to further affirm its safety and efficacy as potential therapeutic candidate.

Thank you for your positive feedback and valuable suggestion. We fully agree that discussing linarin's efficacy in other similar conditions would strengthen our manuscript. In response to your comment, we have revised the Discussion section to include relevant literature on linarin's pharmacological effects in other disease models and its safety profile from chronic administration studies. These additions can be found in the revised manuscript (Lines 424-426, 434-442, and 466-475), where we now provide supporting evidence from recent studies demonstrating linarin's anti-inflammatory properties across different pathological contexts as well as data on its long-term safety. We believe these modifications significantly enhance the translational relevance of our findings while maintaining

focus on acute pancreatitis. We sincerely appreciate your insightful suggestion.

We are grateful for the Editorial Office’s and reviewers' insightful feedback, which guided our revisions. The authors hope the current version addresses all concerns satisfactorily.

Sincerely,

Jie Peng

Department of Gastroenterology, Xiangya Hospital, Central South University

National Clinical Research Center for Geriatric Disorders, Xiangya Hospital, Central South University

Email: pengjie2014@csu.edu.cn
